# Targeting of dermal myofibroblasts through death receptor 5 arrests fibrosis in mouse models of scleroderma

**DOI:** 10.1038/s41467-019-09101-4

**Published:** 2019-03-08

**Authors:** Jong-Sung Park, Yumin Oh, Yong Joo Park, Ogyi Park, Hoseong Yang, Stephanie Slania, Laura K. Hummers, Ami A. Shah, Hyoung-Tae An, Jiyeon Jang, Maureen R. Horton, Joseph Shin, Harry C. Dietz, Eric Song, Dong Hee Na, Eun Ji Park, Kwangmeyung Kim, Kang Choon Lee, Viktor V. Roschke, Justin Hanes, Martin G. Pomper, Seulki Lee

**Affiliations:** 1 Russell H. Morgan Department of Radiology and Radiological Science, Johns Hopkins University School of Medicine, Baltimore, 21205 MD USA; 2 Center for Nanomedicine at the Wilmer Eye Institute, Johns Hopkins University School of Medicine, Baltimore, 21205 MD USA; 3 Theraly Fibrosis Inc., Germantown, 20876 MD USA; 4 Department of Dermatology, Johns Hopkins University School of Medicine, Baltimore, 21205 MD USA; 5 Department of Biomedical Engineering, Johns Hopkins University School of Medicine, Baltimore, 21205 MD USA; 6 Scleroderma Center, Division of Rheumatology, Johns Hopkins University School of Medicine, Baltimore, 21224 MD USA; 7 Division of Pulmonary and Critical Care Medicine, Johns Hopkins University School of Medicine, Baltimore, 21205 MD USA; 8 McKusick-Nathans Institute of Genetic Medicine, Johns Hopkins University School of Medicine, Baltimore, 21205 MD USA; 9 Department of Immunobiology, Yale University School of Medicine, New Haven, 06520 CT USA; 10 College of Pharmacy, Chung-Ang University, Seoul, 06974 Republic of Korea; 11 Biomedical Research Institute, Korea Institute of Science and Technology, Seoul, 02792 Republic of Korea; 12 School of Pharmacy, SungKyunKwan University, Jangangu, 16419 Suwon Republic of Korea; 13 Department of Materials and Science, Johns Hopkins University, Baltimore, 21218 MD USA

## Abstract

Scleroderma is an autoimmune rheumatic disorder accompanied by severe fibrosis in skin and other internal organs. During scleroderma progression, resident fibroblasts undergo activation and convert to α-smooth muscle actin (α-SMA) expressing myofibroblasts (MFBs) with increased capacity to synthesize collagens and fibrogenic components. Accordingly, MFBs are a major therapeutic target for fibrosis in scleroderma and treatment with blocking MFBs could produce anti-fibrotic effects. TLY012 is an engineered human TNF-related apoptosis-inducing ligand (TRAIL) which induces selective apoptosis in transformed cells expressing its cognate death receptors (DRs). Here we report that TLY012 selectively blocks activation of dermal fibroblasts and induces DR-mediated apoptosis in α-SMA^+ ^MFBs through upregulated DR5 during its activation. In vivo, TLY012 reverses established skin fibrosis to near-normal skin architecture in mouse models of scleroderma. Thus, the TRAIL pathway plays a critical role in tissue remodeling and targeting upregulated DR5 in α-SMA^+^ MFBs is a viable therapy for fibrosis in scleroderma.

## Abstract (web summary)

Dermal myofibroblasts are responsible for fibrosis development in scleroderma. Here the authors show that a bioengineered recombinant human TRAIL ligand reverses established fibrosis in mouse models of scleroderma by targeting the death receptor 5 and inducing apoptosis of myofibroblasts.

## Introduction

The critical role of collagen-producing myofibroblasts (MFBs) in tissue remodeling has been widely studied^1–5^. In wound healing, MFBs initiate the scarring process by producing collagens and disappear likely through apoptosis after resolution of the wound^6^. When MFBs differentiated from fibroblasts or stellate cells continue to proliferate, they accumulate at the leading edge of active fibrosis while simultaneously synthesizing and depositing extracellular matrix (ECM), inducing fibrosis in major organs. Overactive MFBs in skin induce fibrosis in scleroderma, either in a diffuse (a.k.a. systemic sclerosis, SSc) or a localized pattern (morphea)^2^. Scleroderma impacts the skin of the most visible body parts such as face and hands and in the diffuse form can lead to severe dysfunction and failure of internal organs, including lungs, heart, kidneys, and stomach, resulting in it having the highest death rate of any rheumatic condition. Since no drugs have emerged for the treatment of scleroderma, although symptomatic relief with immunosuppressants is available, there is a significant unmet need in the treatment of this disease^7^. The current experimental therapeutic strategies under clinical investigation inhibit one of the multiple fibrogenic molecules involved in inflammation and fibroblast activation by utilizing neutralizing antibodies or small molecules that can target transforming growth factor-β1 (TGF-β1)^8^, interleukin-6 (IL-6)^9^, or platelet-derived growth factor receptors (PDGFRs)^10,11^. However, such targeted therapies have not yet reached the level of disease-modifying treatments. Alternatively, we discovered a method to remove excessive MFBs in areas of fibrosis directly, while leaving other normal cells and the process of wound healing unaltered, ameliorating established skin fibrosis in preclinical models of scleroderma.

Previously, we and others reported that human hepatic stellate cells (HSCs) upregulate α-smooth muscle actin (α-SMA) (encoded by the gene *ACTA2*), DR4, and DR5 during activation and become sensitive to TRAIL^12^ through DR-mediated apoptosis in vitro^13,14^. We discovered that TLY012, a PEGylated long-acting recombinant human TRAIL^15^ comprised of an isoleucine zipper amino acid motif that favors trimer formation at the N-terminus with a poly(ethylene glycol) (PEG) molecule, ameliorates liver fibrosis and cirrhosis without off-target toxicity in rat models by selectively depleting α-SMA^+^DR^+^ HSCs^13^. It has been reported that characteristics of activated HSCs resemble those of MFBs^16,17^. Here we show that TLY012 induces DR-mediated apoptosis in α-SMA^+ ^MFBs through upregulated DR5 and reverses established skin fibrosis in scleroderma by targeting α-SMA^+^DR^+ ^MFBs differentiated from dermal fibroblasts, the predominant profibrogenic cell population in skin fibrosis.

## Results

### Dermal MFBs upregulate DR5 and become sensitive to TRAIL-induced apoptosis

**Fig. 1:**
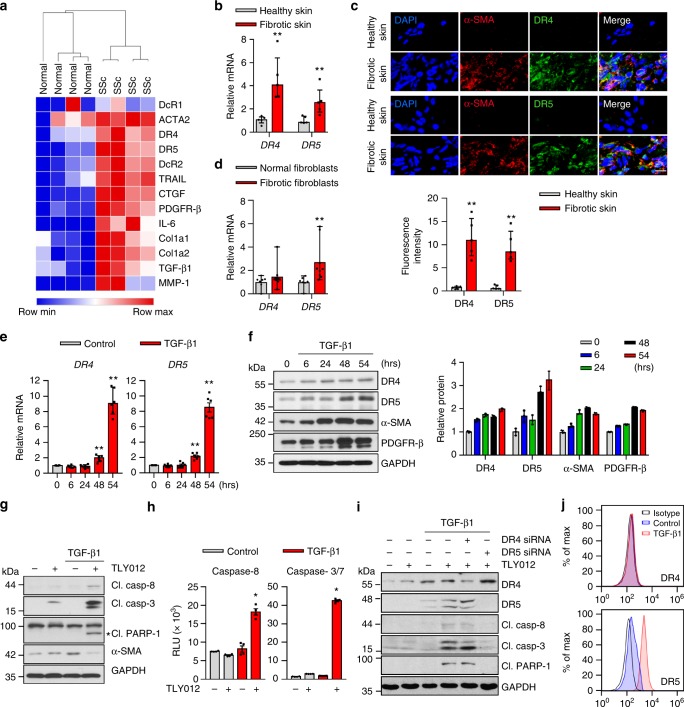
Myofibroblasts (MFBs) differentiated from primary human dermal fibroblasts (HDFs) become sensitive to death receptor (DR)-mediated apoptosis through upregulated DR5. **a** RNA-seq data of skin biopsies of patients with systemic sclerosis (SSc) demonstrated upregulated mRNA *ACTA2*, *DR4*, *DR5*, *TRAIL*, and other fibrogenic components compared to normal skin (*n* = 4 biologically independent samples). **b** Relative mRNA *DR4* and *DR5* expression from the skin of patients with SSc and morphea (*n* = 5 biologically independent samples). **c** Representative double-immunostaining for DR4 (top, green), DR5 (bottom, green), α-SMA (red), and nuclei (DAPI, blue) in the healthy and fibrotic skin samples (scale bars, 10 µm). The fluorescence intensity was measured by ImageJ software (*n* = 5 biologically independent samples). **d** Relative mRNA *DR4* and *DR5* expression from normal and fibrotic fibroblasts isolated from patients (*n* *=* 7 normal fibroblast, *n* = 9 fibrotic fibroblasts biologically independent samples). **e** Relative mRNA *DR4* and *DR5* expression in HDFs treated with TGF-β1 (10 ng/mL) (*n* = 6 biologically independent experiments). **f** Western blot analysis of DR4, DR5, α-SMA, and PDGFR-β in HDFs treated with TGF-β1 (10 ng/mL). Quantification of protein levels normalized to GAPDH (*n* = 3 biologically independent experiments). **g** Representative immunoblots of apoptosis markers cleaved (Cl.) caspase-8, caspase-3, and PARP-1 in TGF-β1 (10 ng/mL for 54 h) activated HDFs treated with TLY012 (1 μg/mL) for 6 h (*n* = 3 biologically independent experiments). **h** Caspase-8 and 3/7 activity in TGF-β1 (10 ng/mL for 54 h) activated HDFs treated with TLY012 (1 μg/mL) for 6 h (*n* = 4 biologically independent experiments). **i** Knockdown effects of DR4 and DR5 on TLY012-induced apoptosis in HDFs activated by TGF-β1 (10 ng/mL for 54 h) as shown by western blot analysis (*n* = 4 biologically independent experiments). **j** Flow cytometry analysis to determine the surface expression of DR4 and DR5 in HDFs activated by TGF-β1 for 54 h (*n* = 3 biologically independent experiments). Data are shown as median ± interquartile range. The Mann–Whitney test was used. *P < 0.05, **P < 0.01, ***P < 0.001, ****P < 0.0001 vs healthy skin or control

We initially investigated whether DRs were upregulated in skin fibrosis to determine their potential as a therapeutic target. We analyzed RNA-seq data from skin biopsies of healthy and SSc patients obtained from a previously published study^18^ and confirmed highly upregulated mRNA *DR4* and *DR5* along with other fibrogenic components, including *ACTA2*, *Col1a2*, *TGF-β1*, and *PDGFR-β*, in skin from patients with SSc (Fig. 1a). Interestingly, endogenous mRNA *TRAIL* is increased in fibrotic skin. Induction of mRNA *DR4*, *DR5*, *TRAIL*, and fibrogenic components in fibrotic skin from patients with SSc and morphea was also determined by real-time quantitative PCR (qPCR) (Fig. 1b and Supplementary Table 1). Furthermore, we observed that DR4 and DR5 are mainly colocalized with α-SMA^+^ cells in the fibrotic skin tissues (Fig. 1c). We also confirmed induction of mRNA *ACTA2, Col1a2, TGF-β1, PDGFR-β, DR4*, and *DR5* expression in dermal fibroblasts isolated from patients with SSc and morphea compared to normal skin (Fig. 1d and Supplementary Table 2). Protein levels for DR4 and DR5 are also increased in fibrotic fibroblasts compared to normal fibroblasts (Supplementary Fig. 1a). These results indicate α-SMA^+^ MFBs may be a viable therapeutic target for skin fibrosis in patients with scleroderma.

To investigate further the ability of TLY012 to induce DR-mediated apoptosis in α-SMA^+^ MFBs differentiated from primary human dermal fibroblasts (HDFs), the levels of DRs, α-SMA, collagens, and PDGFR-β in HDFs were monitored in response to TGF-β1 exposure. TGF-β is upregulated in fibrotic tissues and modulates fibroblast phenotype and function, inducing MFBs trans-differentiation in various organs^19–21^. TGF-β1 significantly induced mRNA and protein levels for DR4 and DR5 along with other fibrogenic molecules in HDFs as validated by qPCR (Fig. 1e and Supplementary Fig. 1b) and western blot (Fig. 1f). When α-SMA^+^DR^+^ MFBs were treated with TLY012 for 6 h, DR-mediated apoptosis was clearly observed in MFBs, but not in quiescent HDFs, in a dose-dependent fashion (Supplementary Fig. 2a, b) as evidenced by increased expression of apoptosis markers, cleaved (Cl.) caspase-8, caspase-3, and PARP-1 (Fig. 1g), as well as increased caspase-8 and caspase-3/7 activities (Fig. 1h). Accordingly, fibrotic fibroblasts from SSc patients were sensitive to TLY012 and humanized anti-DR5 agonistic antibody (1 μg/mL), conatumumab, compared to that of normal fibroblasts (Supplementary Fig. 2c). TRAIL induces apoptosis by binding to two closely related receptors, DR4 and DR5, expressed on the cell surface followed by the recruitment of the adaptor protein, Fas-associated death domain (FADD), and caspase-8 to form the death-inducing signaling complex (DISC)^5^. To investigate the role of each DR on MFB apoptosis, we first attempted to reduce the expression of either DR4 or DR5 in HDFs by using small interfering RNA (siRNA). Surprisingly, TGF-β1-activated MFBs with down-regulated DR5, but not DR4, showed markedly reduced apoptosis after TLY012 treatment (Fig. 1i and Supplementary Fig. 2d). Similarly, conatumumab induced strong apoptosis in MFBs compared to that of humanized anti-DR4 agonistic antibody, mapatumumab (Supplementary Fig. 2e, f). To explain further selective apoptosis through DR5, we characterized the distribution of each DR on cell membranes. Flow cytometry analysis revealed that, although HDFs induce both mRNA and protein DR4 and DR5 levels during activation, DR5 is predominantly expressed on the cell surface (Fig. 1j).

It should be noted that MFB-like cells are difficult to kill^22,23^. For instance, α-SMA^+^ MFBs differentiated from activated HSCs are resistant to cytotoxic drugs (e.g., doxorubicin, cisplatin, H_2_O_2_) mainly due to an upregulation of anti-apoptotic molecules such as BCL-2, BCL-XL, and c-IAP1 as well as c-FLIP. Interestingly, we reported that activated HSCs also induce the pro-apoptotic molecule BAK and reduce expression of the anti-apoptotic molecule XIAP to increase TRAIL sensitivity^6^. In MFBs differentiated from HDFs, we discovered that TGF-β1 induces anti-apoptotic mRNA *BCL-2*, *BCL-XL*, and *MCL-1*, but also substantially upregulates a series of pro-apoptotic mRNAs *BAX*, *BAK, BIM*, *BID*, *BAD*, *PUMA*, and *NOXA* (Supplementary Fig. 3), which are known to increase sensitivity of transformed cells to TRAIL^24^. These results taken together indicate that activated HDFs become sensitive to DR-mediated apoptosis likely due to the combination of increased DR5 density on the plasma membrane coupled with upregulation of selected pro-apoptotic proteins that are associated with TRAIL signaling.

### TLY012 reverses skin fibrosis in mouse models of scleroderma

Human TRAIL is biologically active in murine models, allowing the use of a human protein across species. Unlike humans, mice and rats have only one apoptosis-inducing DR, which is similar to human DR5^25^. As such, TLY012 demonstrated similar receptor binding affinity to both human and mouse DR5 as determined by biolayer interferometry (Supplementary Fig. 4a, b). Next, we examined if TLY012 or mouse anti-DR5 agonistic antibody, MD5-1, selectively induces apoptosis in mouse dermal MFBs. After treatment with TGF-β1, mouse dermal fibroblasts (MDFs) showed increased DR5 levels and became sensitive to TLY012 and MD5-1 (Supplementary Fig. 4c-h). Notably, TLY012 or MD5-1 induced apoptosis was prevented by depletion of DR5 by shRNA or CRISPR in MDFs, (Supplementary Fig. 4e, g, h) indicating that TLY012 induces apoptosis primarily through DR5 in dermal MFBs differentiated from both mouse and human dermal fibroblasts.

**Fig. 2:**
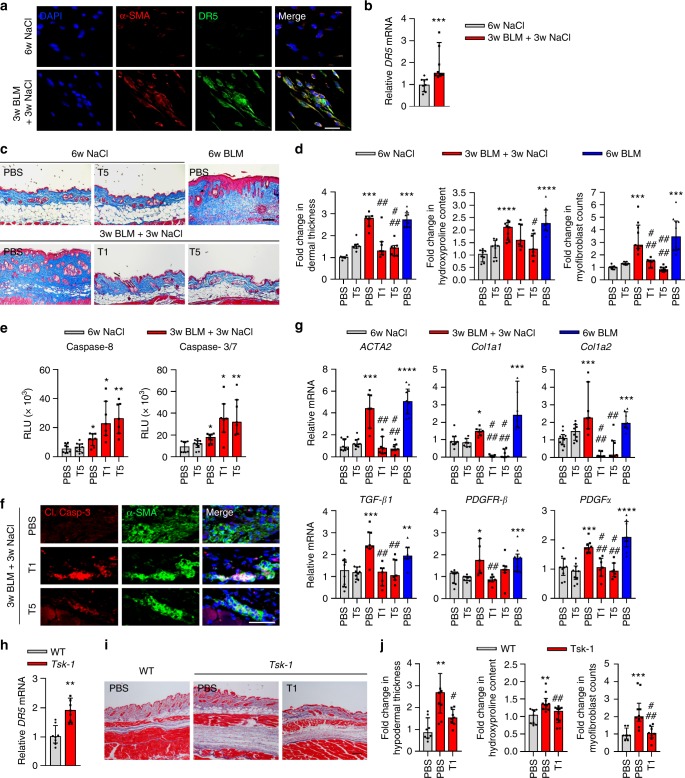
TLY012 reverses skin fibrosis in bleomycin (BLM)-induced and tight skin-1 (*Tsk-1*) transgenic mouse models. **a** Representative double-immunostaining for α-SMA (red), DR5 (green), and nuclei (DAPI, blue) in normal (6w NaCl) and BLM-induced skin fibrosis (3w BLM + 3w NaCl; scale bars, 30 µm). **b** Relative mRNA *DR5* levels in the skin of normal (*n* = 9) and BLM-induced mice (*n* = 10). **c** Representative images of Trichrome-stained sections of control and BLM-induced mice (100 µm). **d** Analysis of dermal thickness (*n* = 7 /group, *n* = 10 6w BLM/PBS), hydroxyproline content (*n* = 8 /group, *n* = 9 3w BLM + 3w NaCl/PBS, 6w BLM/PBS), and myofibroblast counts (*n* = 7 6w NaCl/PBS, T5, 3w BLM + 3w NaCl/T1, *n* = 9 3w BLM + 3w NaCl/T5, *n* = 10 3w BLM + 3w NaCl/PBS, 6w BLM/PBS). **(e)** Measurement of caspase-8 and caspase-3/7 activities (*n* = 8 6w NaCl/PBS, 3w BLM + 3w NaCl/PBS, *n* = 10 6w NaCl/T5, *n* = 7 3w BLM + 3w NaCl/T1, T5). **f** Representative double-immunostaining for cleaved (Cl.) caspase-3 (red) and α-SMA (green) in BLM-induced mice treated with TLY012 (scale bars, 50 µm). **g** Relative mRNA in the skin (*n* = 10 6w NaCl/PBS, T5, *n* = 8 3w BLM + 3w NaCl/PBS, *n* = 7 3w BLM + 3w NaCl/T1, T5, *n* = 10 6w BLM/PBS). **h** Relative mRNA *DR5* levels in the skin of *Tsk-1* mice (*n* = 8 WT, *n* *=* 9 *Tsk-1*). **i** Representative images of Trichrome-stained sections in *Tsk-1* mice (scale bars, 100 µm). **j** Analysis of hypodermal thickness (*n* *=* 8), hydroxyproline content (*n* = 8 WT/PBS, *n* = 10 *Tsk-1*/PBS, TLY012), and myofibroblast counts (*n* = 8 WT/PBS, *Tsk-1*/TLY012, *n* = 10 *Tsk-1*/PBS). *n* biologically independent animals. Data are shown as median ± interquartile range. The Mann–Whitney test was used. *P < 0.05, **P < 0.01, ***P < 0.001, ****P < 0.0001 vs 6w NaCl + PBS or WT + PBS; ^#^*P* < 0.05, ^##^*P* < 0.01, ^###^*P* < 0.001, ^####^*P* < 0.0001 vs 3w BLM + 3w NaCl + PBS or *Tsk-1* + PBS

The anti-fibrotic efficacy of TLY012 was first evaluated in a mouse model of scleroderma that was induced by repeated subcutaneous bleomycin (BLM) injections^26^ in DBA2/J mice. We elected to initiate the treatment of TLY012 three weeks after the continuous BLM treatment, since skin fibrosis in this model is evident at this point. Mice challenged with BLM for the first three weeks followed by NaCl treatment showed similar characteristics of skin fibrosis compared to mice treated with BLM for six weeks. To avoid strong BLM-induced inflammatory responses, the TLY012 treatment was applied in the absence of BLM. Mice received TLY012 for three weeks, a number of histological and biochemical assays were performed, and comparisons were made between vehicle (PBS) and TLY012-treated mice (1 or 5 mg/kg, intraperitoneally (i.p.), every other day) as well as mice continuously treated with BLM without TLY012 for six weeks. As observed in human skin fibrosis, there was substantial DR5 immunoreactivity colocalized to α-SMA^+^ cells in the skin of BLM treated mice (Fig. 2a) along with elevated mRNA *DR5* expression (Fig. 2b). As previously described, BLM treatment markedly induced prominent skin fibrosis with collagen depositions (as determined by trichrome staining and hydroxyproline content), dermal thickening, and the presence of MFB cell populations, which were significantly reduced to near-normal levels by TLY012 (Fig. 2c, d and Supplementary Fig. 5a, b). Furthermore, TLY012 did not negatively impact body weight in mice (Supplementary Fig. 5c). To confirm if TLY012 selectively induced apoptosis in areas of fibrosis, caspase activity was monitored via representative apoptotic markers in skin tissues. We found that TLY012 significantly increased the activity of caspase-8 and caspase-3/7 exclusively in areas of skin fibrosis, but not in the healthy tissues (Fig. 2e). Furthermore, TLY012 treatment induced Cl. caspase-3 immunoreactivity in α-SMA^+^ immunoreactive MFBs in diseased tissues compared to PBS-treated skin fibrosis (Fig. 2f). Taken together, our results suggest that the site of action of TLY012 is likely to be predominant in α-SMA^+^DR5^+^ MFBs localized to areas of fibrosis. Consistent with the results from human skin fibrosis, BLM treatment significantly induced mRNA *ACTA2*, *Col1a1*, *Col1a2*, *TGF-β1*, *PDGFR-β*, and *PDGFα* in fibrotic skins and TLY012 significantly reduced this mRNA induction close to normal levels (Fig. 2g). Interestingly, TLY012 strongly inhibited mRNA *Col1a1/Col1a2* synthesis in skin fibrosis but not in healthy tissues, whereas mRNA *ACTA2,* as well as hydroxyproline content (collagen marker) and number of MFBs, were similar to levels observed in normal skin tissues (Fig. 2d). In addition, no reduction in normal levels of collagen, α-SMA^+^ MFBs, and other biomarkers were observed in normal animals treated with TLY012. This data suggests that TLY012 would not impact crude fibroblast count or related fibroblast activity profiles after removing massively accumulated α-SMA^+^DR5^+^ MFBs in fibrotic areas.

The effects of TLY012 were further evaluated in tight skin-1 (*Tsk-1*) transgenic mice. The *Tsk-1* phenotype is caused by a dominant mutation in the fibrillin-1 gene that leads to scleroderma-like disease with minor infiltrates, autoantibody production, and fibrosis of the skin, mimicking later stages of skin fibrosis with less inflammation^26^. This model exhibits evidence of pathological skin fibrosis at four to five weeks of age. Accordingly, treatment with TLY012 (1 mg/kg, i.p., every other day) in the *Tsk-1* mice was begun at five weeks of age and the outcomes were evaluated at ten weeks. Consistent with BLM-treated fibrotic skin, mRNA *DR5* was increased (Fig. 2h) and DR5 immunoreactivity was colocalized to α-SMA^+^ cells in the skin of ten-week-old *Tsk-1* mice compared to wild-type (WT) skin (Supplementary Fig. 6a). *Tsk-1* mice demonstrated strong skin fibrosis with increased hypodermal thickening, collagen depositions, and MFB accumulation, which was significantly reduced by TLY012 treatment compared to that of healthy skin from WT mice (Fig. 2i, j and Supplementary Fig. 6b, c). Furthermore, TLY012 increased the levels of caspase-8 and caspase-3/7 activities as well as Cl. caspase-3 immunoreactivity in α-SMA^+^ immunoreactive MFBs in diseased tissues when compared to PBS-treated *Tsk-1* mice skin (Supplementary Fig. 6d, e). These results indicated that TLY012 could potentially reverse established skin fibrosis.

### DR5 is a therapeutic target for skin fibrosis

**Fig. 3:**
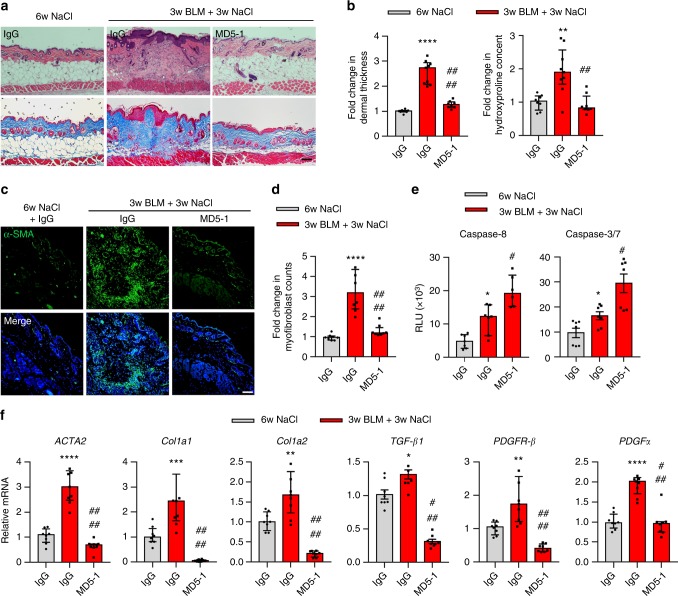
Mouse anti-DR5 antibody (MD5-1) reverses skin fibrosis in bleomycin (BLM)-induced mouse model of skin fibrosis. DBA2/J mice were divided into three groups: the control group treated with NaCl (s.c., day 0–42) and IgG (5 mg/kg, i.p., every other day, day 22–42, *n* = 8) and the BLM-induced groups treated with BLM (s.c., day 0–21) and IgG (5 mg/kg, i.p., every other day, day 22–42, *n* = 9) or MD5-1 (5 mg/kg, i.p., every other day, day 22–42, *n* = 8). **a** Representative images of H&E and Trichrome-stained skin sections (*n* = 8 mice treated with NaCl/IgG, *n* = 9 BLM/IgG, *n* = 8 BLM/MD5-1, biologically independent animals; scale bars, 100 µm). **b** Analysis of dermal thickness and hydroxyproline content. **c** Representative immunostaining for α-SMA (green) and nuclei (DAPI, blue) (*n* = 8 biologically independent animals; scale bars, 50 µm). **d** Myofibroblast counts in the skins of control and BLM-induced groups (*n* = 8 biologically independent animals). **e** Measurement of caspase-8 and caspase-3/7 activities in the skins of control and BLM-induced groups (*n* = 7 biologically independent animals). **f** qPCR analysis of mRNA *ACTA2*, *Col1a1*, *Col1a2*, *TGF-β1*, *PDGFR-β,* and *PDGFα* in the skin (*n* = 8 biologically independent animals). Data are shown as median ± interquartile range. The Mann–Whitney test was used. *P < 0.05, **P < 0.01, ***P < 0.001, ****P < 0.0001 *vs* 6w NaCl + IgG; ^#^*P* < 0.05, ^##^*P* < 0.01, ^###^*P* < 0.001, ^####^*P* < 0.0001 vs 3w BLM + 3w NaCl + IgG

To confirm DR5 as a therapeutic target for skin fibrosis in mice, mouse anti-DR5 agonistic antibody (MD5-1) was administered to mice with BLM-induced skin fibrosis and compared to those treated with control antibody immunoglobulin G (IgG). Consistent with the TLY012 results, MD5-1 (100 μg per mouse, i.p., every other day) significantly normalized BLM-induced dermal thickening, hydroxyproline content, and MFB accumulation (Fig. 3a–d). Accompanying with the reduced skin fibrosis was the induction of caspase-8 and caspase-3/7 activity (Fig. 3e) and marked reduction of mRNA *ACTA2*, *Col1a1*, *Col1a2*, *TGF-β1*, *PDGFR-β*, and *PDGFα* (Fig. 3f) in areas of skin fibrosis. MD5-1 also strongly inhibited mRNA *Col1a1*/*Col1a2* and *TGF-β1* expression compared to normal skin tissue, however, mRNA *ACTA2* and MFBs counts as well as hydroxyproline levels maintained comparable to normal skin tissue as observed in TLY012 studies.

We failed to demonstrate the efficacy of MD5-1 in *Tsk-1* mice because of the hepatotoxicity of MD5-1 in C57BL/6 mice. It has been reported that MD5-1 induces hepatotoxicity in a mouse strain-dependent manner, resulting in cholestatic liver injury in C57BL/6 (B6) mice^27,28^ but not in DBA2/J mice (Supplementary Fig. 7 and Supplementary Fig. 8a). *Tsk-1* mice have a B6 genetic background, potentially making them susceptible to the hepatotoxic effect of MD5-1. To further investigate the potential toxicity of TLY012 and MD5-1 in B6 mice, mice were treated with either TLY012 (5 mg/kg) or MD5-1 (100 µg per mouse) via i.p. injection, every other day, for three weeks. Mice treated with MD5-1 showed severe weight loss from day 12 and developed jaundice with increased liver injury as evidenced by elevated aspartate aminotransferase (AST) and alanine aminotransferase (ALT) levels (Supplementary Fig. 8b–e). In contrast, TLY012 did not show any weight loss or liver toxicity in B6 mice (Supplementary Fig. 8b–e). Unlike MD5-1, TLY012 demonstrated no strain-dependent hepatotoxicity in mice.

### TGF-β signaling regulates the DR5 expression in dermal MFBs

**Fig. 4:**
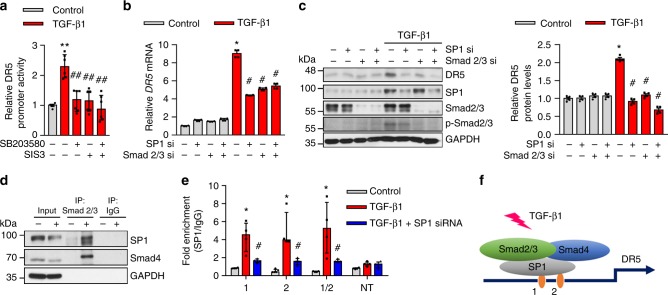
TGF-β signaling regulates the DR5 expression in HDFs though Smad2/3-SP1 complexes. **a** Measurements of DR5 promoter activity with the dual-luciferase-reporter system in HDFs treated with SB203580 (10 μM, Smad2 inhibitor) and SIS3 (5 μM, Smad3 inhibitor) for 2 h followed by TGF-β1 (10 ng/mL) treatment for 54 h (*n* = 6 biologically independent experiments). **b**, **c** HDFs were transfected with SP1 and/or Smad 2/3 siRNA for 24 h. The cells were then exposed to TGF-β1 for 54 h. **b** mRNA *DR5* levels in siRNA transfected HDFs with and without TGF-β1 treatment (*n* = 4 biologically independent experiments). **c** Effects of siRNA-mediated knockdowns of SP1 and Smad2/3 on TGF-β1-induced DR5 levels in HDFs by western blot. Relative DR5 protein levels normalized to GAPDH (*n* = 4 biologically independent experiments). **d** Western blot analysis of Smad2/3 immunoprecipitates (IP) of HDFs with or without TGF-β1 treatment (*n* = 3 biologically independent experiments). **e** Binding assessment with ChIP assays of the two potential SP1 sites (site 1: −195 to −190, site 2: −159 to −154) and non-target site (NT) to the DR5 promoter in HDFs treated with TGF-β1 (*n* = 4). **f** A representation of TGF-β pathway including Smad2/3, Smad4, and SP1 complexes in the translation mechanism of DR5. Data are shown as median ± interquartile range. The Mann–Whitney test was used. *P < 0.05, **P < 0.01 vs control; ^#^*P* < 0.05, ^##^*P* < 0.01 vs with TGF-β1

Understanding the function of DR5 and transcription factors essential for the regulation of DR5 gene expression in MFBs may provide insight regarding the overall utility of this target to treat scleroderma and other fibrosis-related diseases. The transcription factors for upregulation of DR expression in cancer cells have been reported^29,30^, however, the mechanisms underlying these actions in MFBs differentiated from HDFs are unknown. TGF-β has long been considered as a key mediator of fibrosis in various organs including skin^31^. Therefore, it is conceivable that a TGF-β dependent pathway is in part or fully responsible for DR5 upregulation. Downstream signals of TGF-β include a family of bifunctional, transcription activators known as Smads^32^. To understand better DR5 gene regulation by TGF-β, HDFs were transfected with a human DR5 promotor-luciferase reporter gene. Inhibition of TGF-β signaling by Smad2 and/or Smad3 inhibitors, SB203580 and SIS3, respectively, strongly prevented DR5 promotor activity as determined by a reporter gene assay (Fig. 4a). In addition to Smad signaling, non-Smad TGF-β pathways are implicated in scleroderma^33^. We investigated whether the non-Smad pathways are involved in DR5 regulation in dermal MFBs while focusing on mitogen-activated protein kinase (MAPK) dependent pathways by treating TGF-β1 activated HDFs with a MAPK inhibitor, PD98059. PD98059 inhibited MAPK related molecules including p-JNK, p-p38 MAPK, and p-p44/42 MPAK, however, it did not prevent TGF-β1 mediated DR5 upregulation (Supplementary Fig. 9a, b). The human DR5 promotor has no Smad binding elements but in silico analysis revealed two potential binding sites (-198 to -116) for SP1^34^. Smads have been shown to complex with SP1 and regulate TGF-β1 induced gene expression^20^. To validate the binding specificity of SP1 and Smad2/3 to the DR5 promoter region, we tested whether knockdown of SP1 or Smads would reduce their association. siRNA-mediated knockdown of Smad2/3 or SP1 reduced the stimulatory effects of TGF-β1 on DR5 expression as determined by qPCR and western blot (Fig. 4b, c). Likewise, knockdown of Smad2/3 by shRNA also decreased TGF-β1 mediated DR5 regulation (Supplementary Fig. 9c, d). Co-immunoprecipitation demonstrated that TGF-β1 stimulated the interaction of Smad2/3 and Smad4 with SP1 in TGF-β1-activated HDFs (Fig. 4d). The chromatin immunoprecipitation (ChIP) assay further validated that TGF-β1 induced complexes of Smad2/3 and Smad4 as well as increased binding to the SP1 binding site of the DR5 promoter, whereas siRNA-mediated knockdown of SP1 led to decreased binding (Fig. 4d, e). These findings demonstrate that TGF-β1 induces DR5 likely by canonical Smad signaling and the transcription factor SP1 (Fig. 4f). The human DR4 promotor has three potential binding sites (−606 to −20) for SP1. We further studied the binding specificity of SP1 and Smads to the DR4 promotor regions by ChIP assay as described above and confirmed that TGF-β1 induces DR4 as seen in the case of DR5 (Supplementary Fig. 10), although DR4 demonstrated limited expression on the surface of TGF-β1-activated HDFs.

## Discussion

The pathogenic mechanisms of underlying skin fibrosis in scleroderma are largely unknown, however, MFBs are thought to be one of the significant originators of this disorder. During disease progression, resident fibroblasts transformed into proliferative, fibrogenic, and contractile α-SMA^+^ MFBs with increased capacity to synthesize ECM and multiple fibrogenic components in order to orchestrate skin fibrogenesis. By nature, dermal MFBs are a major therapeutic target for skin fibrosis in scleroderma, however, the lack of robust strategies to selectively reverse or deplete this phenotype in vivo hampers this approach. Here we observed that selective depletion of excessive α-SMA^+^DR5^+^ MFBs accumulated in areas of fibrosis by externally supplied DR5 agonists, TLY012 and anti-DR5 antibody, reversed skin fibrosis to near-normal skin architecture without notable toxicity in animal models with active, progressive skin fibrosis. Furthermore, we discovered that increased DR5 expression associated with TGF-β signaling is a hallmark of dermal MFBs and a viable therapeutic target for anti-fibrotic therapy.

Due to their unique ability to induce apoptosis in cancer cells in vitro while showing no apparent toxicity to normal cells, recombinant human TRAIL and DR4/5 agonistic antibodies have been widely studied for cancer therapy^35^. Clinical studies of TRAIL-based therapy revealed a broad tolerability in humans but failed to demonstrate a robust therapeutic benefit in oncology, possibly due to the short half-life of recombinant TRAIL and/or heterogeneity and TRAIL resistance in primary cancer cells. TLY012 is a long-acting version of recombinant human TRAIL and provides the same beneficial biological functions of endogenous TRAIL while overcoming the inherent short half-life of the protein. TRAIL resistance in a range of cancer cells is likely due to a combination of multiple factors including lack of surface DR4 or DR5 that trigger DR-mediated apoptosis as well as elevated anti-apoptotic proteins and/or the absence of pro-apoptotic proteins that play key roles in TRAIL resistance and sensitization, respectively. We found that dermal MFBs become extremely sensitive to DR-mediated apoptosis, possibly due to upregulated DRs and substantially increased expressions of multiple pro-apoptotic proteins during fibroblast activation. While TRAIL initiates apoptosis in cancer cells via binding to both DR4 and DR5 expressed on the cellular membrane, in dermal MFBs we observed that, although fibroblast activation induces both DR4 and DR5 at the mRNA and protein levels, DR5 is predominantly expressed on the cellular membrane and increased MFB susceptibility to TRAIL-induced apoptosis. Scleroderma fibroblasts are highly heterogeneous and likely to behave differently from dermal MFBs of healthy fibroblasts. To prove clinical transferability and relevance, we tested whether DR5 agonists induce apoptosis in fibroblasts from patients with SSc and morphea. Unlike many TRAIL-resistant tumor cells, scleroderma fibroblasts were sensitive to both TLY012 and humanized anti-DR5 agonistic antibody-induced apoptosis. We showed elevated DR5 levels in a limited number of fibrotic skin tissues and scleroderma fibroblasts, however, to address clinical heterogeneity of scleroderma and dermal MFB properties, this should be further validated in an expanded number of clinical samples. We further investigated the molecular regulators and transcription factors responsible for the increased gene expression of DR5 and revealed that TGF-β/Smad is a major pathway which plays an important role in the regulation of DR5. Although TGF-β is a key mediator of fibrogenesis that modulates various fibroblast phenotypes and functions, fibroblasts can also be activated and differentiated by other molecules such as platelet-derived growth factor (PDGF). Thus, different pathways involved in fibroblast activation need to be studied further to understand the mechanisms of how MFBs become sensitive to TRAIL-induced apoptosis during the progression of fibrosis in vivo.

Our hypothesis of directly targeting MFBs by TRAIL as a potential therapeutic approach for fibrosis was investigated in two well-established, inducible (BLM) and genetic (*Tsk-1*), mouse models of skin fibrosis. The BLM-induced model resembles early and inflammatory stages of skin fibrosis whereas the *Tsk-1* model mimics later and non-inflammatory stages. In both models, the population of α-SMA^+^DR5^+^ MFBs was significantly increased in fibrotic skin areas and selective induction of apoptosis of these cells by systemically administered DR5 agonists at pharmacologically relevant doses effectively ameliorated skin fibrosis. Importantly, TLY012 demonstrated superior anti-fibrotic efficacy in both models when the treatment was initiated after fibrosis had already established. This implies that TLY012 can potentially treat patients with advanced skin fibrosis and differs from other experimental drugs that are aimed to suppress inflammation or inhibit fibroblast proliferation. Such drugs would be effective in the early stage of fibrosis but not in the late stage with advanced fibrosis being associated with the less inflammatory process and a large number of persistent MFBs. Both animal models are useful for testing drug candidates, however, it does not fully address the etiology of scleroderma. Studying the role of TRAIL in additional mouse models which represent different features of scleroderma, such as autoimmunity and vasculopathy, warrant further investigation.

Interestingly, we validated that expression of endogenous TRAIL was increased in skin tissues collected from patients with SSc and morphea, further supporting several sets of data that have shown elevated levels of circulating TRAIL in scleroderma patients^36^. We hypothesize that the body inherently elevates TRAIL levels during the progression of fibrosis to remove abnormally accumulated MFBs, contributing to tissue remodeling. This could be a potential mechanism of how excessive MFBs are removed from the body through apoptosis since normal dermal fibroblasts are resistant to TRAIL but become sensitive to TRAIL-induced apoptosis when the cells differentiate into MFBs. However, during the progression of fibrogenesis or in the advanced fibrosis stage, the elevated levels of endogenous TRAIL are not sufficient enough to deplete highly proliferative and massively accumulated persistent MFBs, suggesting the need for an external source of TRAIL. In this perspective, recombinant human TRAIL analogs like TLY012 could be a potential candidate for human testing since TLY012 can remove persistent MFBs in a natural way similar to endogenous TRAIL in the body.

Our study supports this hypothesis as we provide evidence that TLY012 reverses established fibrosis in preclinical models. It is obvious that TRAIL signaling and DRs play critical roles in tissue remodeling and the pathology of scleroderma. MFB persistence is a major characteristic of fibrosis and effective targeting of excess MFBs differentiated from various source cells in organ fibrosis is widely considered a therapeutic strategy to control fibrosis in different organs including skin^2^, liver^17^, and kidney^37^. Based on the observations reported here along with those from our previous report^6^, TLY012 may have broad anti-fibrotic properties in a variety of diseases characterized by and involving MFBs. Since human TRAIL is considered generally safe in patients with no dose-limiting or major organ toxicity, TLY012 could offer improved therapeutic opportunities for the treatment of advanced fibrosis. Further research into combination regimens, TLY012 combined with a drug that targets different aspects of the fibrotic cascade, could also represent potent therapeutic effects.

## Methods

### RNA-seq analysis

RNA-seq data of control skin biopsies and skin biopsies of SSc patients were obtained from a previously published study with the accession ID of SRP037553^18^. Single 4 mm punch biopsies were obtained from lesional forearm skin of four patients with early, diffuse SSc within 6 months of the first onset of non-Raynaud’s symptoms and normal skin of four controls without disease. Kallisto^38^ was used to align to the genome and to calculate the estimated counts. Sleuth was utilized to calculate the differential expression profiles of the genes. First, the transcriptome index was created using the ENSEMBL hg38 rel 89 genome with a k-mer value of 31. Paired-end reads were analyzed with Kallisto settings accounting for read bias (--bias) with 100 bootstraps (-b 100) and single paired reads were analyzed with –l 200 and –s 20, which were average estimated lengths of the paired reads. TPM values generated by Kallisto were used for the heatmap. Each value was first added to one to avoid non-real numbers when computing the binary logarithm (Log2) of the values. Raw TPM values for each sample and the differential expression between control versus SSc samples are included in Supplementary Data 1.

### Patients

Skin biopsies were obtained from 5 female patients with SSc or morphea. The average age of the patients was 55.4 years old (range 33–68 years). Healthy skin samples were obtained from unaffected tissue of patients. Participants were recruited from the Cutaneous Translational Research Program (CTREP), Department of Dermatology of Johns Hopkins University School of Medicine and the study protocol was approved by the Johns Hopkins Medicine Institutional Review Board. We have compiled all relevant ethical regulations (declaration of Helsinki) and written informed consent was obtained from all study subjects. Detailed patient information is summarized in Supplementary Table 1.

### Cell culture

Human primary dermal fibroblasts (HDFs) (American Type Culture Collection (ATCC); Manassas, VA, USA) were maintained in fibroblast basal medium (ATCC) supplemented with fibroblast growth kit low serum (ATCC)^39^. Cells were incubated with recombinant human TGF-β1 (10 ng/mL; R&D Systems, Minneapolis, MN, USA) and a combination of the following: TLY012^13^, anti-DR4 Ab (Mapatumumab; Creative Biolabs, Shirley, NY, USA), anti-DR5 Ab (Conatumumab; Creative Biolabs), Smad 2 inhibitor SB203580 (Selleckchem, Houston, TX, USA), Smad3 inhibitor SIS3 (Selleckchem), or MAPK inhibitor PD98059 (Selleckchem). For gene silencing studies, cells were transfected with DR4 siRNA (Santa Cruz Biotechnology, Inc., Santa Cruz, CA, USA), DR5 siRNA (Santa Cruz Biotechnology, Inc), Smad2/3 siRNA (Santa Cruz Biotechnology, Inc.), SP1 siRNA (Santa Cruz Biotechnology, Inc.) or Smad2/3 shRNA (Santa Cruz Biotechnology, Inc.) using Lipofectamine 2000 (Invitrogen, Carlsbad, CA, USA) according to the manufacturer’s protocol. Human dermal fibroblasts from patients were generated as previously described^20^. Skin biopsies from three patients were digested using dispase II (Sigma-Aldrich, St. Louis, MO, USA) with 10% heat-inactivated FCS and cells were maintained in DMEM/F-12 medium. All cell lines were negative for mycoplasma.

### Caspase activity assay

TLY012-induced apoptosis signaling was quantified using the Caspase-Glo 3/7 and Caspase-Glo 8 assay kit (Promega Corporation, Madison, WI, USA) and detected by a multiple microplate reader (Bio-Tek Instruments Inc, Winooski, VT, USA).

### DR5 lentiviral transduction

Mouse dermal fibroblasts (MDFs; ScienCell, Carlsbad, CA, USA) were plated in 100 mm culture dishes 1 day prior to transduction. 10 µg/mL of polybrene (Millipore) was treated before lentiviral particle infection. 2 × 10^5^ infectious units of virus (IFU) of mouse DR5 shRNA (m) lentivirus (Santa Cruz Biotechnology, Inc.) or control shRNA lentivirus (Santa Cruz Biotechnology, Inc.) was added to the cells and incubated for 48 h with or without mouse TGF-β1 (10 ng/mL; R&D Systems) for activation. Cells were either collected for analysis of knockdown or plated on to 96 well plates. Caspase 3/7 activity was measured after 3 h of TLY012 incubation.

### DR5 CRISPR knockout

Mouse dermal fibroblasts (MDFs; ScienCell) were transfected with control donor or mouse DR5 knockout guide RNA (Tnfrsf10b Mouse Gene Knockout kit, CRISPR, Origene Technologies KN517973) and Lipofectamine 3000 (Thermo Fisher Scientific Inc., Wilmington, DE, USA) as manufacturer’s protocol. Briefly, the cells were split 1:10 48 h post transfection and grown an additional 3 days. The cells split 7 more times for 3 weeks and puromycin (2 μg/mL) was treated. Finally, single cells were isolated using 96 well plate and knockout was confirmed using western blotting. Typically, 10^4^ cells were plated on 96 well plate and were serum starved for 24 h before TLY012 or DR5 antibody treatment.

### Cell viability assay

Cells were incubated with the CellTiter-Glo Luminescent Cell Viability Assay kit (Promega Corporation) for 10 min and detected using a multiple microplate reader (Bio-Tek Instruments Inc).

### Animal studies

The animal experiments followed the Guide to the Care and Use of Animals laboratory animal manual of the National Institute of Health, and they were approved by the Johns Hopkins Medical Institute Animal Care and Use Committee. To evaluate the effects of TLY012 and anti-DR5 Ab (MD5-1; Bio X cell, West Lebanon, NH, USA) on established skin fibrosis in vivo, bleomycin (BLM, Enzo Life Sciences, Inc., Farmingdale, NY, USA) was injected in 6-weeks-old female DBA2/J mice as previously described^40,41^. Briefly, mice received subcutaneous injection of 100 μL BLM (0.5 mg/mL) dissolved in 0.9% NaCl in a single location on the upper back every other day to induce dermal fibrosis for 3 or 6 weeks. DBA2/J mice were divided into two control groups, NaCl (s.c., day 0–42, *n* = 10) and PBS or TLY012 (5 mg/kg, i.p., every other day, day 22–42, *n* = 10). Three BLM-induced fibrosis groups (3w BLM + 3w NaCl) were treated with BLM (s.c., day 0-21, *n* = 10) and NaCl (s.c., day 22–42) with PBS (i.p., *n* = 10) or TLY012 (1 or 5 mg/kg, i.p., every other day, day 22–42, T1 and T5, respectively, *n* = 10). An additional group was treated with BLM (s.c., day 0-42) for six weeks (*n* = 10). PBS or TLY012 (5 mg/kg) treated groups with subcutaneous injections of 100 μL of 0.9% NaCl served as control. For MD5-1 treatment, BLM-challenged mice for 3 weeks were treated with MD5-1 (100 μg per mouse), or IgG (Bio X cell, 100 μg per mouse) by intraperitoneal (i.p.) injections every other day. After 3 weeks, the mice were sacrificed by cervical dislocation. In the tight skin-1 (*Tsk-1*) model of SSc^42^, three groups of mice were used for this experiment: wild-type (WT) treated with PBS (*n* = 8) *Tsk-1* mice treated with PBS (*n* = 10) or TLY012 (1 mg/kg, *n* = 10) for five weeks. TLY012 treatment was started at the age of five weeks and the outcome was evaluated at the age of ten weeks. For toxicity tests in C57BL/6 (B6) mice, 8-weeks mixed gender B6 mice were treated with TLY012 (5 mg/kg) or MD5-1 (100 µg per mouse) by i.p. injection every other day for 3 weeks (*n* = 5). In all animal studies, mice were randomized into treatment groups.

### Immunofluorescence

Skin samples were fixed in 4% paraformaldehyde and embedded in paraffin. The 5 μm sections were blocked with BlockAid™ blocking solution (Invitrogen) for 1 h and incubated with antibodies against DR4 (Abcam, Cambridge, MA, USA), DR5 (Abcam), α-smooth muscle actin (α-SMA; Sigma-Aldrich), and cleaved Caspase-3 (Cell Signaling Technology, Danvers, MA, USA) at 4 °C overnight. Alexa Flour 488 (Invitrogen), Alexa Flour 594 (Invitrogen), or Cy5 (Invitrogen) conjugated antibodies were used as secondary antibodies and sections were further incubated for 1 h at room temperature (RT). Cell nuclei were visualized with DAPI (Vector Laboratories, Burlingame, CA, USA). Stained sections were analyzed using a Zeiss LSM710 confocal microscope (Carl Zeiss, Göttingen, Germany). Myofibroblast (α-SMA positive cell) counts were manually measured at four randomly selected sites at 200x magnification in a blinded manner by two independent examiners.

### Quantitative real time-PCR

Total RNAs were isolated from skin tissues and HDFs using RNeasy mini kit (Qiagen Sciences, Inc., Germantown, MD, USA). The concentration of total RNAs was measured using an UV-Vis spectrophotometer (NanoDrop2000, Thermo Fisher Scientific Inc.) and reverse-transcribed with a high capacity cDNA reverse transcription kit (Applied Biosystems, Carlsbad, CA, USA). Gene expression was quantified by SYBR green real-time PCR on a StepOnePlus^TM^ system (Applied Biosystems). The primer sequences are listed in Supplementary Table 3. Data were analyzed according to the comparative Ct method. Glyceraldehyde 3-phosphate dehydrogenase (GAPDH) or 18sRNA was used to normalize for the amounts of cDNA within each sample.

### Western blot analysis

Proteins were extracted from HDFs by RIPA buffer (Thermo Fisher Scientific Inc.). Extracted proteins were separated by SDS/PAGE and subsequently transferred to PVDF membranes (Bio-Rad, Hercules, CA, USA). Membranes were blocked in 3% BSA for 1 h at RT and incubated with primary antibodies against DR4 (Abcam), DR5 (Abcam), α-SMA (Sigma-Aldrich), PDGFR-β (Santa Cruz Biotechnology, Inc.), cleaved Caspase-8 (Cell Signaling Technology), cleaved Caspase-3 (Cell Signaling Technology), cleaved PARP-1 (Cell Signaling Technology), SP1 (Cell Signaling Technology), Smad2/3 (Cell Signaling Technology), Smad4 (Cell signaling Technology), p-Smad2/3 (Cell signaling Technology), p-JNK (Cell signaling Technology), p-p38 MAPK (Cell signaling Technology), p-p44/42 MAPK (Cell signaling Technology) and GAPDH (Santa Cruz Biotechnology, Inc.) overnight at 4 °C, followed by incubation with HRP-conjugated secondary antibodies (Thermo Fisher Scientific) for 1 h at RT. The antibodies used in this study are summarized in Supplementary Table 5. Proteins were visualized using ECL Western Blotting Substrate (Promega Corporation). Quantification was performed with ImageJ software (National Institutes of Health).

### Histological analysis

Fibrotic skin biopsies were obtained from the BLM-injected sites on mice. Paraffin-embedded sections (5 μm) were stained with hematoxylin and eosin or Masson’s trichrome to evaluate collagen type I deposition (Abcam). All sections were examined on a Nikon Eclipse E600 microscope with DS-Fi2 digital camera (Nikon, Melville, NY, USA) at ×100 and ×200 magnifications. Dermal and hypodermal thickness was measured at four randomly selected sites in each mouse in a blinded manner by two independent examiners.

### Hydroxyproline analysis

Total collagen content of skin tissue samples was quantified using a hydroxyproline assay kit (Chondrex, Inc., Redmond, WA, USA) according to the manufacturer’s instructions. Briefly, 5 N HCl solution was added to skin tissue, followed by overnight hydrolysis at 120 °C. The processed tissue samples were incubated with chloramine-T solution for 20 min at RT. Each sample was treated with DMAB solution and incubated for 30 min at 60 °C for color development. Samples were read in a 96-well plate at 560 nm on a multiple microplate reader (Bio-Tek Instruments Inc.).

### Serum analysis

Serum samples were collected from mice on Day 21 after TLY012 or MD5-1 injection. ALT and AST levels were measured by an automated clinical chemistry analyzer, Vet Axcel (Alfa Wassermann, West Caldwell, NJ, USA).

### Co-immunoprecipitation (CoIP)

HDFs were lysed by IP lysis buffer (25 mM Tris, 150 mM NaCl, 0.05% NP-40) and 10% lysates were used as input. Cell lysates were incubated with Smad2/3 (Cell Signaling) or normal IgG (Thermo Fisher Scientific) antibodies for 2 h at 4 °C followed by overnight incubation at 4 °C with Protein A/G PLUS-Agarose (Santa Cruz Biotechnology, Inc.). Unbound proteins were washed using washing buffer (150 mM NaCl, 0.05% NP-40) and bound protein complexes were analyzed by western blot.

### Chromatin immunoprecipitation (ChIP) assays

ChIP assays were performed using the EpiTect ChIP OneDay kit (Qiagen Sciences, Inc.). Cells were cross-linked with fixing buffer at 37 °C for 10 min and sonicated using a Branson CPX2800 ultrasonic bath (Branson Ultrasonics, Danbury, CT). ChIP ready chromatin was incubated with antibodies against SP1 (Cell Signaling Technology) or normal rabbit IgG antibodies (Santa Cruz Biotechnology, Inc.) with Protein A beads. After DNA isolation and purification, ChIP DNA samples were determined by quantitative real time-PCR with the primers summarized in Supplementary Table 4.

### Reporter assay

Cells were transfected with DR5 promoter-pGL2 constructs (Addgene, Cambridge, MA, USA) and pRL-SV40P (Addgene) expressing renilla luciferase using Lipofectamine 2000 (Invitrogen). After 24 h incubation, cells were treated with 10 μM Smad2 inhibitor (SB203580, Selleckchem) and 5 μM Smad3 inhibitor (SIS3, Selleckchem) for 2 h before TGF-β1 treatment. Luciferase activity was analyzed using a Dual-luciferase-Reporter system (Promega Corporation) with a luminometer (Berthold Technologies, Oak Ridge, TN, USA). Luciferase activity was normalized by renilla luciferase.

### Flow cytometry

For analysis of DR4 or DR5 surface expression, HDFs were washed three times in PBS and incubated with PE-conjugated anti-human DR4 (eBioscience, Inc., San Diego, CA, USA), DR5 (eBioscience, Inc.), or isotype IgG1 (eBioscience, Inc.) antibodies. Stained cells were measured by an Accuri C6 plus flow cytometer (BD Biosciences, San Jose, CA, USA).

### Binding affinity assay

Binding affinity measurement was performed using an Octet RED96 System (ForteBio, Menlo Park, CA, USA). Anti-Human IgG Fc Capture biosensors (ForteBio) were used to immobilized rhTRAIL-R2/Fc and rmTRAIL-R2/Fc chimera proteins (R&D systems). Biosensors were hydrated in the kinetic buffer for 10 min prior to protein immobilization. rhTRAIL-R2/Fc and rmTRAIL-R2/Fc chimera proteins were immobilized for 50 s. Then, the sensors were washed with kinetic buffer for 60 s and reacted with various concentrations (from 6.25 to 100 nM) of TLY012 for 200 s association steps. After association, the sensors were moved to the kinetic buffer for dissociation steps. The association and dissociation data were corrected by the baseline response in the kinetic buffer and the equilibrium dissociation constant (*K*_D_) was calculated using Octet Data Analysis software (ForteBio).

### Statistical analysis

Statistical analysis was performed using GraphPad Prism 7 (GraphPad Software, San Diego, CA, USA). Statistics were presented as median ± interquartile range (IQR) with at least three independent studies. Representative morphological images were taken out of at least three studies with parallel results. Student’s *t*-test (unpaired, two-tailed) was used for analysis of two-group comparison. If normality was rejected by D’Agostino-Pearson normality test, Mann–Whitney *U*-test was applied for nonparametric data. *P-*values less than 0.05 were considered significant.

### Reporting summary

Further information on experimental design is available in the Nature Research Reporting Summary linked to this article.

## Supplementary information

Supplementary Information

Description of Additional Supplementary Files

Supplementary Data 1

Reporting Summary

## Data Availability

The data that support the findings of this study are available from the corresponding author upon reasonable request.
