# Supplementary material for: Targeting of dermal myofibroblasts through death receptor 5 arrests fibrosis in mouse models of scleroderma
Source: Nat Commun. 2019 Mar 8;10:1128. doi: 10.1038/s41467-019-09101-4 (PMC6408468; doi:10.1038/s41467-019-09101-4)

Supplementary Information

Targeting of dermal myofibroblasts through death receptor 5 arrests fibrosis in mouse models of scleroderma

Jong-Sung Park,[1,2,*] Yumin Oh,[1,2,*] Yong Joo Park,[1,2] Ogyi Park,[1,2,12] Hoseong Yang,[3] Stephanie Slania,[4] Laura K. Hummers,[5] Ami A. Shah,[5] Hyoung-Tae An,[1,2] Jiyeon Jang,[1,2] Maureen R. Horton,[6] Joseph Shin,[7] Harry C. Dietz,[7] Eric Song,[8] Dong Hee Na,[9] Eun Ji Park,[9] Kwangmeyung Kim,[10] Kang Choon Lee,[11] Viktor V. Roschke,[12] Justin Hanes,[2,4] Martin G. Pomper,[1,13] and Seulki Lee.[1,2,13]

[1]Russell H. Morgan Department of Radiology and Radiological Science, [2]Center for Nanomedicine at the Wilmer Eye Institute, [3]Department of Dermatology, [4]Department of Biomedical Engineering, [5]Scleroderma Center, Division of Rheumatology, [6]Division of Pulmonary and Critical Care Medicine, [7]McKusick-Nathans Institute of Genetic Medicine, Johns Hopkins University School of Medicine, Baltimore, Maryland, USA

[8]Department of Immunobiology, Yale University School of Medicine, New Haven, Connecticut, USA

[9]College of Pharmacy, Chung-Ang University, Seoul, Republic of Korea

[10]Biomedical Research Institute, Korea Institute of Science and Technology, Seoul, Republic of Korea

[11]School of Pharmacy, SungKyunKwan University, Jangangu, Suwon, Republic of Korea

[12]Theraly Fibrosis Inc., Germantown, Maryland, USA

[13]Department of Materials and Science, Johns Hopkins University, Baltimore, Maryland, USA

*These authors contributed equally.

Correspondence should be addressed to:

Seulki Lee, Ph.D.

The Russell H. Morgan Department of Radiology and Radiological Science, The Center for Nanomedicine at the Wilmer Eye Institute, Department of Materials and Science,

Johns Hopkins University, 400 North Broadway, Baltimore, MD 21287, USA

E-mail: seulki@jhmi.edu

Supplementary Figure 1.

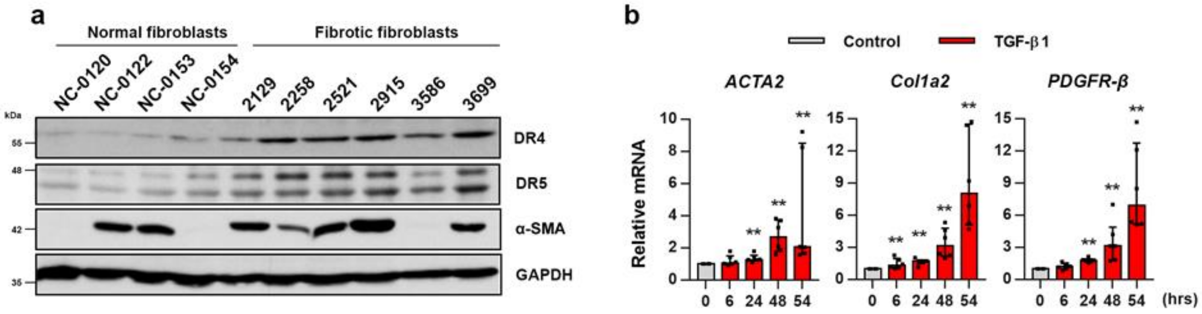

Supplementary Figure 1. Protein and mRNA levels of fibrogenic molecules in fibrotic fibroblasts from patients and TGF-β1 induced human dermal fibroblasts (HDFs). (a) Western blot analysis of DR4, DR5, and α-SMA in dermal fibroblasts from patients. (**b**) qPCR analysis of mRNA *ACTA2*, *Col1a2*, and *PDGFR-β* in HDFs treated with TGF-β1 (10 ng/mL; n = 6). Data are shown as median ± IQR. The Mann-Whitney test was used. **P < 0.01 vs Control

Supplementary Figure 2.

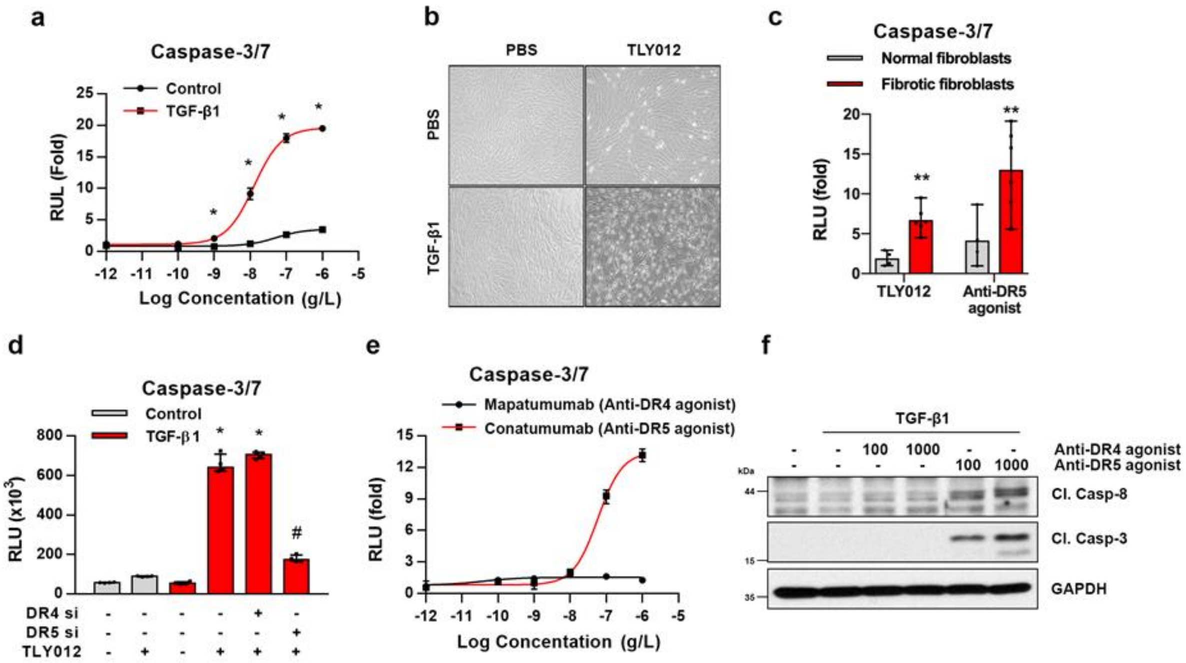

Supplementary Figure 2. TLY012 induces selective DR5-mediated apoptosis in TGF-β1-activated HDFs and fibrotic fibroblasts from patients. (**a-b**) HDFs were exposed to TGF-β1

(10 ng/mL) for 48 hrs. The cells were then treated with various does of TLY012 for 6 hrs. (a) Dose-dependent induction of caspase-3/7 activities in TGF-β1-activated HDFs treated with TLY012 (n = 4). (**b**) Representative images of HDFs incubated with or without TGF-β1 and TLY012 (n = 3; 10x magnification). (c) Caspase-3/7 activities in normal and fibrotic fibroblast isolated from patients treated with TLY012 (1 μg/mL) or humanized anti-DR5 agonistic antibody, conatumumab (1 μg/mL), with Protein G for 6 hours (n = 4-6). (**d**) Caspase-3/7 activity of siRNA-mediated knockdown of DR4 and DR5 on TLY012-induced apoptosis in HDFs activated by TGF-β1 (n = 4). (**e**) Dose-dependent induction of caspase-3/7 activities in TGF-β1-activated HDFs treated with anti-DR5 antibody, conatumumab, and anti-DR4 antibody, mapatumumab (n = 3). (**f**) Western blot analysis of cleaved (Cl.) caspase-8, caspase-3 and GAPDH (n = 3). Data are shown as median ± IQR. Mann-Whitney was used for all. *$P < 0.05$, **$P < 0.01$ vs Control, $^{\#}P < 0.05$ vs TGF-β1.

Supplementary Figure 3.

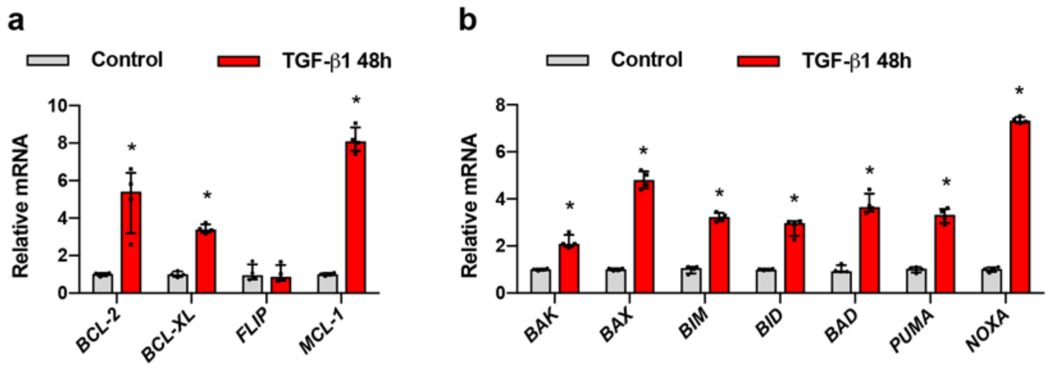

Supplementary Figure 3. mRNA levels of apoptotic signaling molecules in TGF-β1 induced HDFs. qPCR analysis of (**a**) anti-apoptotic and (**b**) pro-apoptotic molecules in HDFs treated with TGF-β1 (10 ng/mL) for 48 hrs *(n* = 4). Data are shown as median ± IQR. Mann-Whitney was used for all. *$P < 0.05$ vs Control.

Supplementary Figure 4.

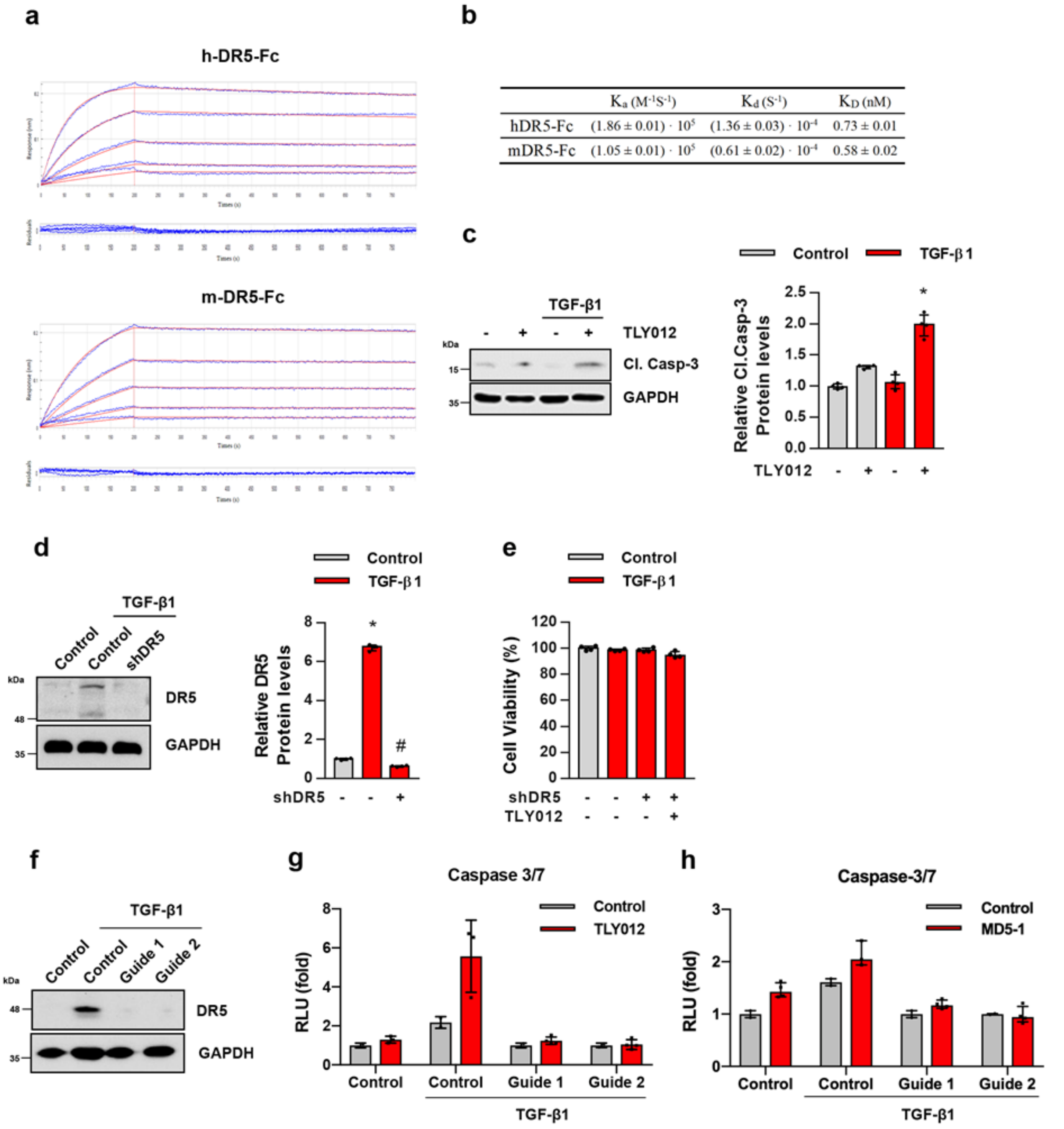

Supplementary Figure 4. Binding affinity of TLY012 against human and mouse DR5 and effects of TLY012 and MD5-1 on mouse dermal fibroblasts (MDFs). (**a**) The death receptors, hDR5-Fc (human, upper) or mDR5-Fc (mouse, lower), were immobilized onto a biosensor followed by incubation with five concentrations of TLY012 (6.25 - 100 nM; bottom to top). The data was analyzed by global fitting using 1:1 binding model. Experimental and fitted curves are indicated by the blue and red lines, respectively, with residuals of the fitted curves shown below

each sensorgram (*n* = 3). (**b**) Kinetic rate constants of TLY012 binding to hDR5 and mDR5. Association (Ka) and dissociation (Kd) rate constant values were calculated by global fitting and KD values were calculated by dividing Kd/Ka (*n* = 3). (**c**) Representative immunoblots and densitometric analysis of Cl. Caspase-3 and GAPDH in mouse TGF-β1 (10 ng/mL for 48 hrs) activated MDFs treated with TLY012 (5 μg/mL) and 10% mouse serum for 24 h (*n* = 4). (**d**) MDFs were transfected with control shRNA lentivirus or mouse DR5 shRNA lentivirus and incubated with or without mouse TGF-β1 for 48 hrs. Protein levels of DR5 and GAPDH were assessed by western blot analysis (*n* = 4). (**e**) Effects of shRNA-mediated knockdown of DR5 on TLY012-induced apoptosis in MDFs activated by TGF-β1. Cell viability was measured with CellTiter-Glo Luminescent cell viability assay kit (*n* = 4). (**f**) Tnfrsf10b (DR5) CRISPR knockout MDFs incubated with or without TGF-β1 for 48 hours. Protein levels for DR5 and GAPDH were assessed by western blot analysis. (**g-h**) Effects of Tnfrsf10b CRISPR knockout of DR5 on (**g**) TLY012-induced or (**h**) MD5-1-induced apoptosis in TGF-β1-activaed MDFs. Cell viability was measured with CellTiter-Glo luminescent cell viability assay kit (*n* = 2-4). Data are shown as median ± IQR. Mann-Whitney was used for all. *$P < 0.05$ vs Control

Supplementary Figure 5.

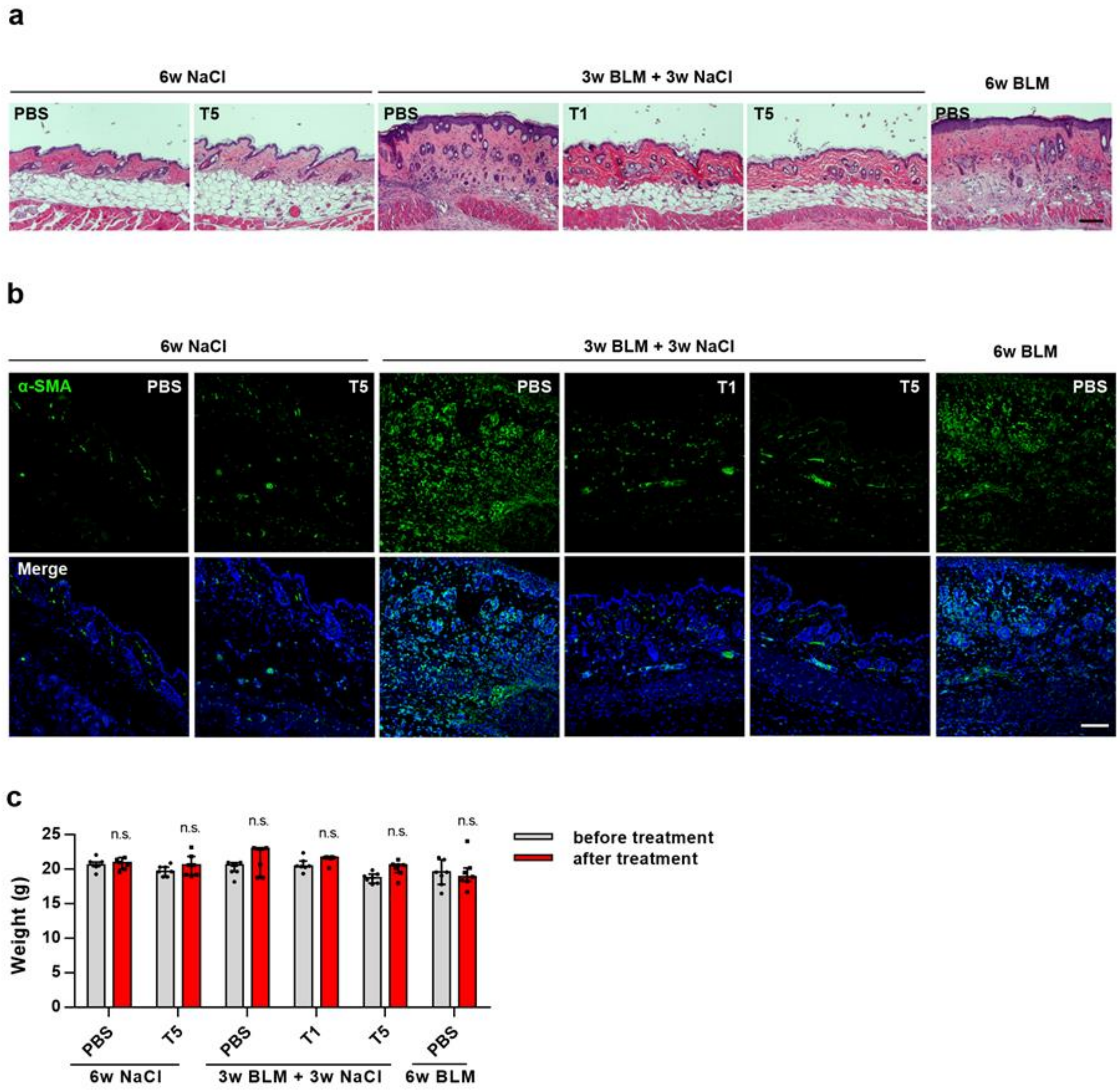

Supplementary Figure 5. TLY012 ameliorates skin fibrosis in BLM-induced skin fibrosis. (a) Representative images of H&E-stained sections of control and treatment groups (*n* = 7-9; scale bars, 100 µm). (b) Representative images of skin sections stained for α-SMA (green) and nuclei (DAPI, blue) (n = 7-10; scale bars, 50 µm). (c) Mice body weight measurements before and after TLY012 treatment (*n* = 7-10). Data are shown as median ± IQR. The Mann-Whitney test was used. n.s. not significant.

Supplementary Figure 6.

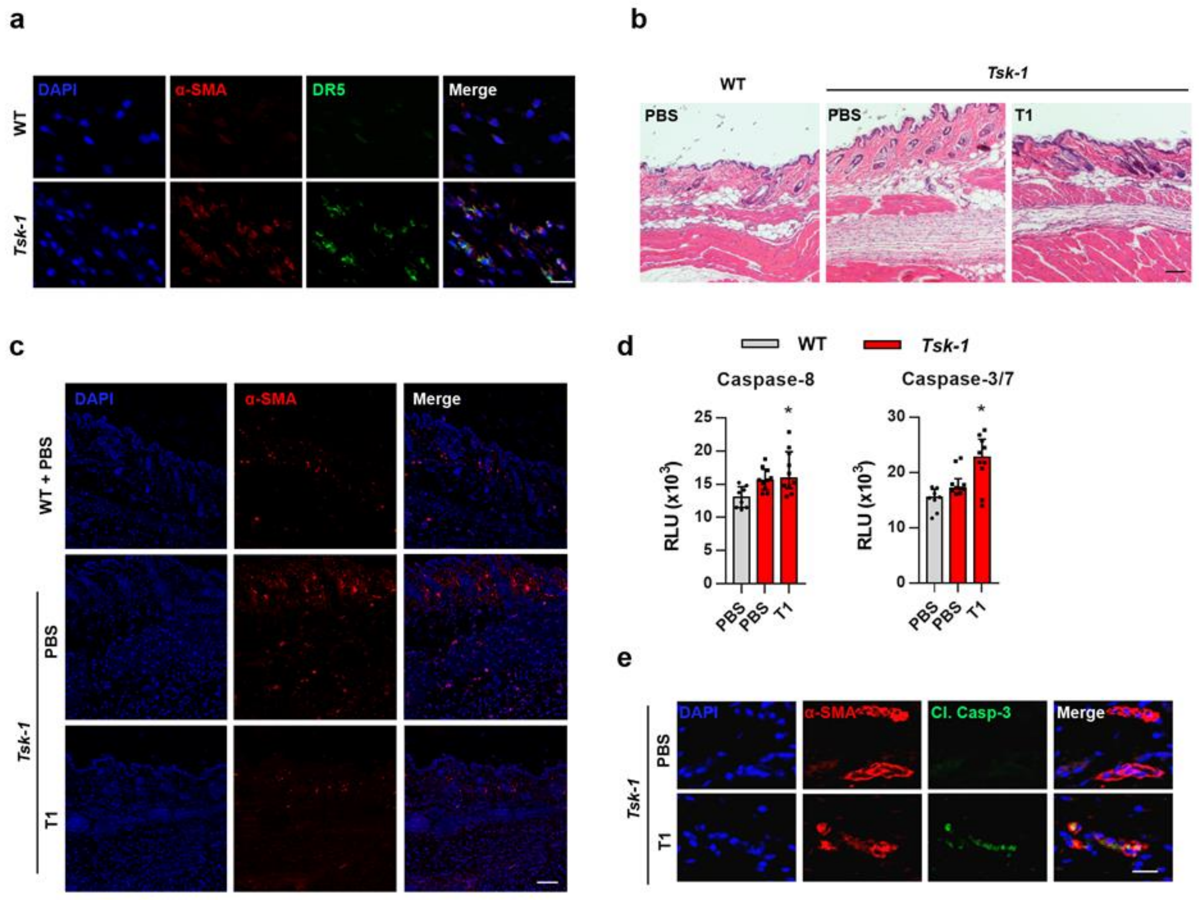

Supplementary Figure 6. Effects of TLY012 on *Tsk-1* mice. (**a**) Representative double-immunostaining for α-SMA (red), DR5 (green), and nuclei (DAPI, blue) in WT and *Tsk-1* (*n* = 4; scale bars, 20 µm). (**b**) Representative images of H&E-stained sections of control and treatment groups (*n* = 8-9; scale bars, 100 µm). (**c**) Representative images of skin sections stained with α-SMA (red) and DAPI (blue) (*n* = 8-9; scale bars, 100 µm). (**d**) Measurement of caspase-8 and caspase-3/7 activities in the skins of WT and *Tsk-1* mice treated with TLY012 (*n* = 8-10). (**e**) Representative double-immunostaining for α-SMA (red) and cleaved (Cl.) caspase-3 (green) in *Tsk-1* mice treated with PBS and TLY012 (*n* = 6; scale bars, 20 µm). Data are shown as median ± IQR. Mann-Whitney was used. *$P < 0.05$.

Supplementary Figure 7.

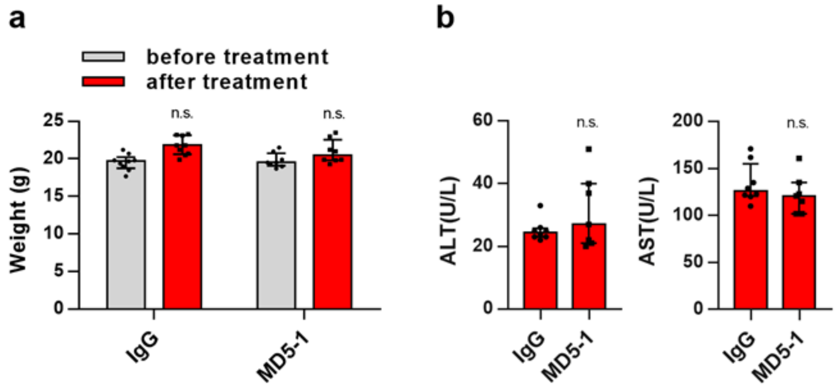

Supplementary Figure 7. MD5-1 demonstrates no adverse effects in the DBA2/J mice with BLM-induced skin fibrosis. (**a**) Mice body weight measurements before and after MD5-1 treatment (*n* = 8-9). (**b**) ALT and ALS levels on BLM-induced mice treated with MD5-1 (*n* = 7-8). Data are shown as median ± IQR. Mann-Whitney was used. n.s. not significant.

Supplementary Figure 8.

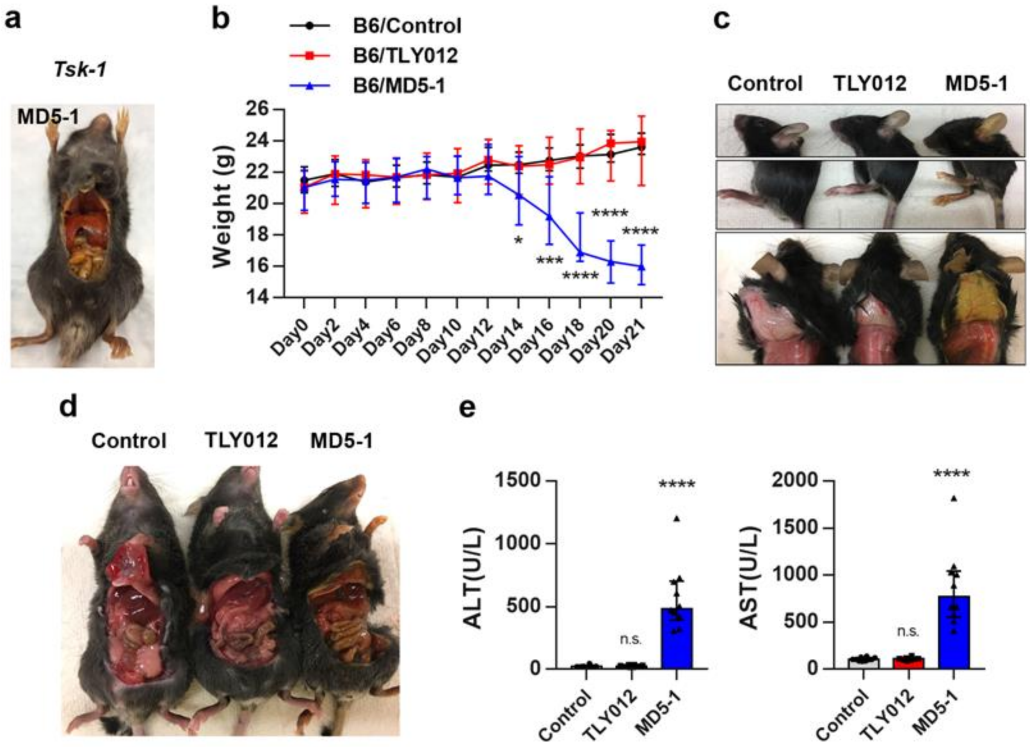

Supplementary Figure 8. Hepatotoxic effects of MD5-1 in *Tsk-1* and C57BL/6 mice. (**a**) Representative photo of severe jaundice development in MD5-1 treated *Tsk-1* mice. *Tsk-1* mice were treated with MD5-1 (100 µg per mouse) by i.p. injection every other day (n = 5). (**b-e**) Mixed gender 8 weeks old C57BL/6 (B6) mice were treated with saline, TLY012 (5 mg/kg), or MD5-1 (100 µg) by i.p. injection every other day for 3 weeks. (**b**) Body weight changes (n = 10). (**c**) Representative photos of jaundice induced by MD5-1 treatment. (**d**) Representative photos of liver injury induced by MD5-1 treatment. (**e**) ALT and AST levels in the serum (n = 8-10). Data are shown as median ± IQR. Mann-Whitney test was used. *P < 0.05, ***P < 0.001, ****P < 0.0001 vs B6/Control. n.s. not significant.

Supplementary Figure 9.

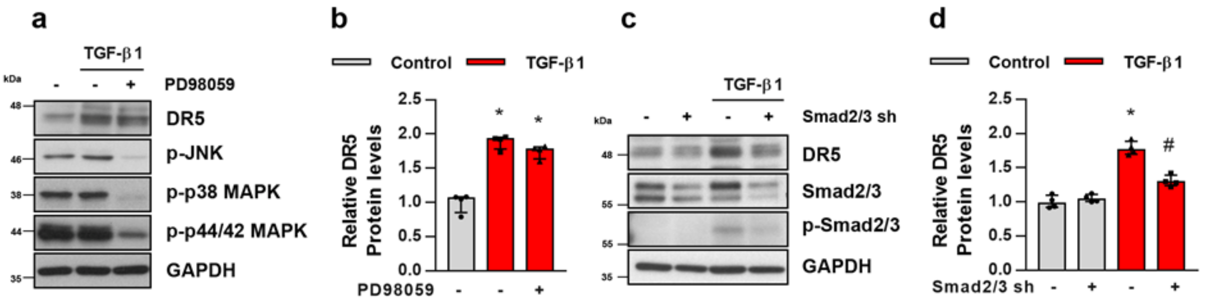

Supplementary Figure 9. DR5 regulation of MAPK pathway inhibition and shRNA-mediated Smad2/3 knockdown in TGF-β1 activated HDFs. (**a-b**) HDFs treated with PD98059 (25 μM, MAPK inhibitor) for 2 hrs followed by TGF-β1 (10 ng/mL) treatment for 54 hrs. (**a**) Western blot analysis of DR5, p-JNK, p-p38 MAPK, p-p44/42 MAPK, and GAPDH (n = 4). (**b**) Densitometric analysis of DR5 shown as relative protein expression normalized to GAPDH (n = 4). (**c-d**) HDFs were transfected with Smad 2/3 shRNA for 24 hrs. The cells were then exposed to TGF-β1 for 54 hrs. (**c**) Expression of DR5, Smad2/3, p-Smad 2/3, and GAPDH assessed by western blot (n = 4). (**d**) Relative DR5 protein levels normalized to GAPDH (n = 4). Data are shown as median ± IQR. The Mann-Whitney test was used. *P < 0.05 vs Control; $^{\#}P$ < 0.05 vs with TGF-β1.

Supplementary Figure 10.

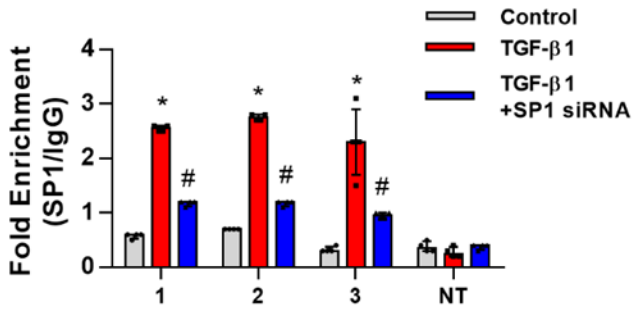

Supplementary Figure 10. ChIP assay assessment of binding of SP1 sites of the human DR4 promotor by complexes of SP1, Smad2/3, and Smad4 in TGF-β1 activated HDFs with or without

siRNA-mediated knockdown of SP1 ($n = 4$). Data are shown as median ± IQR. The Mann-Whitney test was used. *$P < 0.05$ vs Control; $^{\#}P < 0.05$ vs with TGF-β1.

Supplementary Data 1. Skin RNA-seq data. Data is provided in the form of raw TPM values.

Supplementary Table 1. mRNA expressions of death receptors and fibrotic markers in the healthy and fibrotic skin samples from patients with morphea and SSc. Real-time PCR analysis of the fibrotic and healthy human skin samples. 18sRNA was used to normalize for the amounts of cDNA.

Human skin tissues of SSc and Morphea

**Case**	**Sex**	**Age**	**Race**	**FDX**
1156179613	F	64	W	Control and Morphea
1156682343	F	61	W	Control and SSc
1165029641	F	68	B	Control and Morphea
1167040671	F	33	W	Control and Morphea
1248984720	F	51	B	Control and Morphea

**Gene**	**Fibrotic skin** (M): Morphea, (S): SSc				
	1 (M)	2 (S)	3 (M)	4 (M)	5 (M)
DcR1	3.55	8.35	16.56	4.65	6.36
ACTA2	3.06	8.35	1.21	2.22	3.03
DR4	3.08	7.86	3.05	4.07	4.94
DR5	1.47	4.58	1.98	2.68	2.56
DcR2	6.09	2.07	1.55	1.60	1.15
TRAIL	2.52	5.76	5.26	8.29	114.11
CTGF	1.89	2.70	2.74	27.08	29.20
PDGFR-β	3.24	2.93	1.44	2.86	0.34
IL-6	1.47	4.29	2.64	3.31	21.67
Col1a2	3.98	3.62	4.01	10.46	1.42
TGF-β1	2.73	3.02	2.56	4.83	0.03
MMP-1	0.99	106.50	51.26	15.92	34.03

Supplementary Table 2. mRNA expressions of death receptors and fibrotic markers in the normal and fibrotic fibroblasts from patients with morphea and SSc. Real-time PCR analysis of the fibrotic and normal fibroblasts from human skin. 18sRNA was used to normalize for the amounts of cDNA.

Human dermal fibroblasts of SSc and Morphea

**Cell line ID**	**Sex**	**Age**	**Race**	**FDX**
1156179613	F	64	W	Control and Morphea
1156682343	F	61	W	Control and SSc
1165029641	F	68	B	Control and Morphea
Nc-0120	M	51	W	Control
Nc-0122	F	60	W	Control
Nc-0153	M	46	W	Control
Nc-0154	F	55	W	Control
2129	M	55	W	SSc
2258	M	76	W	SSc
2521	F	35	W	SSc
2915	F	58	W	Morphea
3586	F	65	W	Morphea
3699	F	64	W	SSc

**Gene**	**Fibroblast from** (M): Morphea, (S): SSc								
	1 (M)	2 (S)	3 (M)	4 (S)	5 (S)	6 (S)	7 (M)	8 (M)	9 (S)
DcR1	1.38	2.2	0.5	0.86	0.47	0.84	0.56	0.35	2.00
ACTA2	7.3	73.4	24.3	1.68	5.58	1.26	1.4	0.87	1.08
DR4	4.0	1.2	0.4	1.59	1.18	1.23	1.31	1.06	1.28
DR5	5.8	5.5	2.7	2.17	1.59	1.45	1.95	1.21	2.12
DcR2	1.1	1.6	0.8	0.69	1.44	1.01	0.79	1.41	1.01
CTGF	1.47	41.5	4.2	2.17	2.10	1.89	3.06	4.82	2.26
PDGFR-β	5.9	4.9	1.4	1.95	2.01	1.64	3.68	2.78	2.37
Col1a2	3.2	1.4	1.2	2.73	2.22	1.57	3.28	1.68	3.00
TGF-β1	5.0	2.2	1.4	1.52	2.83	2.42	1.93	1.89	1.58

Supplementary Table 3. Sequence of PCR primers used in quantitative Real-time PCR.

Human		
**Gene**	**Forward**	**Reverse**
DR4	TGT GAC TTT GGT TGT TCC GTT GC	ACC TGA GCC GAT GCA ACA ACA G
DR5	AAG ACC CTT GTG CTC GTT GT	AGG TGG ACA CAA TCC CTC TG
DcR1	AAA GTT CCT GCA CCA TGA CC	TGG CAC CAA ATT CTT CAA CA
DcR2	GCT GAA GGG TGT CAG AGG AG	AGC CTG CCT CAT CTT CTT CA
ACTA2	CCA GAG CCA TTG TCA CAC AC	CAG CCA AGC ACT GTC AGG
Col1a2	AGC AGG TCC TTG GAA ACC TT	GAA AAG GAG TTG GAC TTG GC
TGF-β1	CTT CCA GCC GAG GTC CTT	CCC TGG ACA CCA ACT ATT GC
CTGF	CAA GGG CCT CTT CTG TGA CT	ACG TGC ACT GGT ACT TGC AG
TRAIL	TGG CAA CTC CGT CAG CTC GTT	AGC TGC TAC TCT CTG AGG ACC
PDGFR-β1	CAG GAG AGA CAG CAA CAG CA	AAC TGT GCC CAC ACC AGA AG
IL-6	AAT TCG GTA CAT CCT CGA CGG	TTG GAA GGT TCA GGT TGT TTT CT
MMP-1	GAG CTC AAC TTC CGG GTA GA	CCC AAA AGC GTG TGA CAG TA
18sRNA	CTA CCA CAT CCA AGG AAG CA	TTT TTC GTC ACT ACC TCC CCG
GAPDH	AAT CCC ATC ACC ATC TTC CA	TGG ACT CCA CGA CGT ACT CA
**Mouse**		
DR5	TTG GAA TGG CTG GTG TAG TC	TGC TGC TTG CTG TGC TAC
DcR1	TTT CCG GAA TCA TGC CGC CCA	AGG ACC AGC CAG TTT CTG GGA TTT G
DcR2	TGT CCC GCT GGT GAA TAC TGG T	GGA TTC GCA GGG CGC CTT G
ACTA2	GTT CAG TGG TGC CTC TGT CA	ACT GGG ACG ACA TGG AAA AG
TGF-β1	TGA CGT CAC TGG AGT TGT ACG G	GGT TCA TGT CAT GGA TGG TGC
Col1a1	TTC GGA CTA GAC ATT GG	GGG TTG TTC GTC TGT TTC
Col1a2	CCG TGC TTC TCA GAA CAT CA	CTT GCC CCA TTC ATT TGT CT
PDGFR-β1	TGG CCT CTG AGG ACT AAA GC	AAC AGA AGA CAG CGA GGT GG
PDGFα	CTC TTG GAG ATA GAC TCC GTA GG	ACT TCT CTT CCT GCG AAT GG
GAPDH	TTG ATG GCA ACA ATC TCC AC	CGTCCC GTA GAC AAA ATG GT

Supplementary Table 4. Sequence of PCR primers used in ChIP assay.

**DR4**		
**Target**	**Forward**	**Reverse**
Site 1	CGC TTG TAA TCC CAG CAC TTT G	TTT GGC CAG GCT GGT CTC
Site 2	GAG GCT GAG GCA GGA AA	CAG GCT GGA GTG CAG TG
Site 3	GTT CAG GGT TAG CCA ACA GG	ACT TCG CAT TCG GAG TTC AG
Non-target (NT)	CGT TTC TCC ATG TCG GTC AG	GCC TGT AAT CCC AGC ACT TT
**DR5**		
Site 1	CTG TGG TGG AAT TTG CAG TTG	CTG TAC TTT CAC TGC CTC CG
Site 2	CGG AGG CAG TGA AAG TAC AG	CCT TAA AGT AGA TCG GGC ATC G
Site 1/2	GTT GCA CAT TGG ATC TGA TTC G	TTA TGT GTC CAG GCT GAC TTG
Non-target (NT)	CAA GAC CCT GTT CAC AGC CA	GGA AAT GAG TTG AGG GAG GCA

Supplementary Table 5. Antibodies used in this study.

**Antibodies**	**Source/Cat. No./Ref.**	**Host**	**Dilution**
DR4	Abcam (ab8414)	Rabbit	1:1000 (WB), 1:200 (IHC)
DR5	Abcam (ab8416)	Rabbit	1:1000 (WB), 1:200 (IHC)
α-smooth muscle actin (α-SMA)	Sigma (A2547)	Mouse	1:2000 (WB), 1:400 (IHC)
Cl. Caspase-3 (Asp 175)	Cell signaling (9664)	Rabbit	1:1000 (WB), 1:1000 (IHC)
Caspase-8 (1C12)	Cell signaling (9746)	Mouse	1:1000 (WB)
Cl. PARP-1	Cell signaling (9541)	Rabbit	1:1000 (WB)
PDGFR-β1	Santa Cruz (sc-432)	Rabbit	1:1000 (WB)
Smad2/3 (D7G7)	Cell signaling (8685)	Rabbit	1:1000 (WB), 1:100 (IP)
Smad4 (D3M6U)	Cell signaling (38454)	Rabbit	1:1000 (WB)
SP1	Cell signaling (5931)	Rabbit	1:1000 (WB), 1:200 (ChIP)
p-Smad2/3	Cell signaling (8828)	Rabbit	1:1000 (WB)
p-JNK	Cell signaling (9251)	Rabbit	1:1000 (WB)
p-p38 MAPK	Cell signaling (4511)	Rabbit	1:1000 (WB)
p-p44/42 MAPK	Cell signaling (4370)	Rabbit	1:1000 (WB)
GAPDH	Santa Cruz (sc-32233)	Mouse	1:10000 (WB)

Uncropped images

Fig. 1f

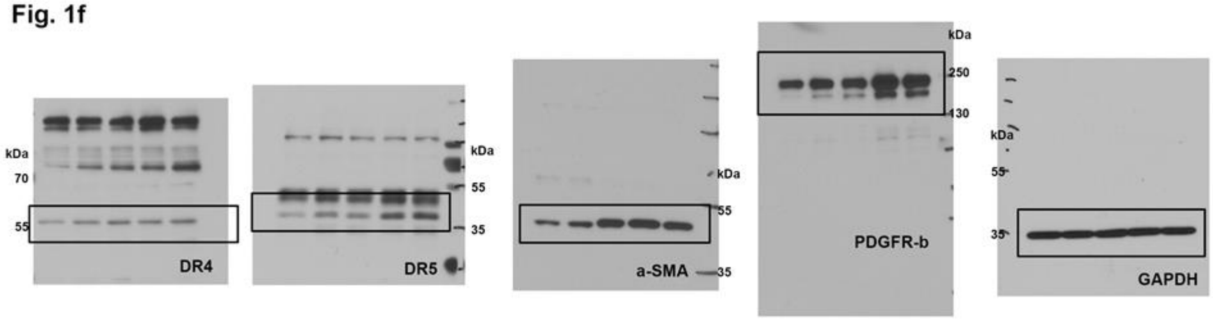

Fig. 1g

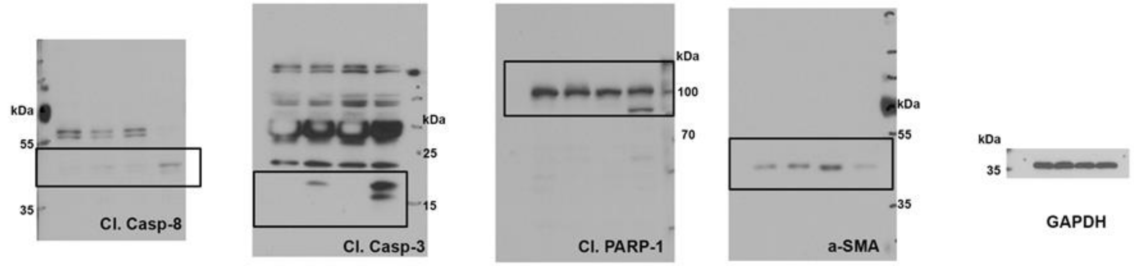

Fig.1 i

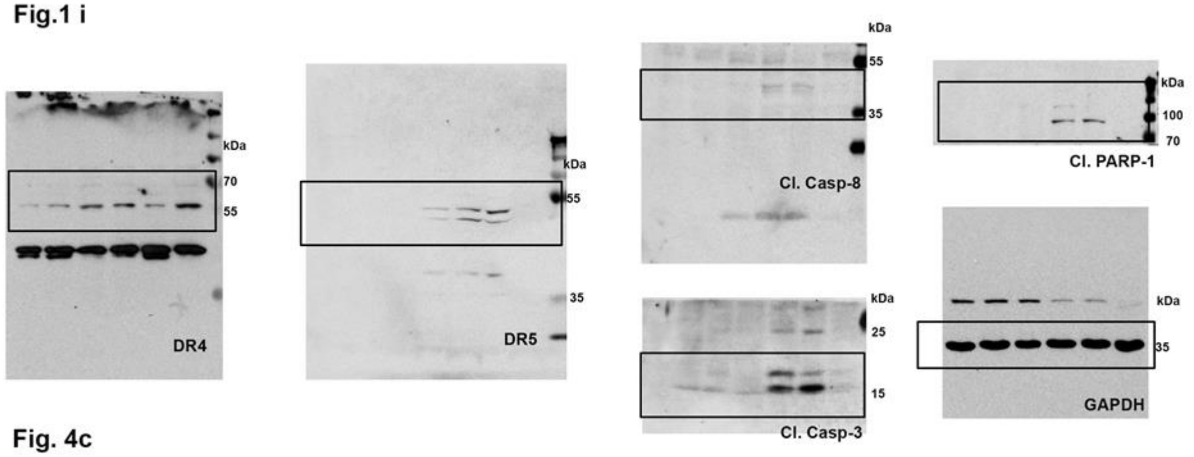

Fig. 4c

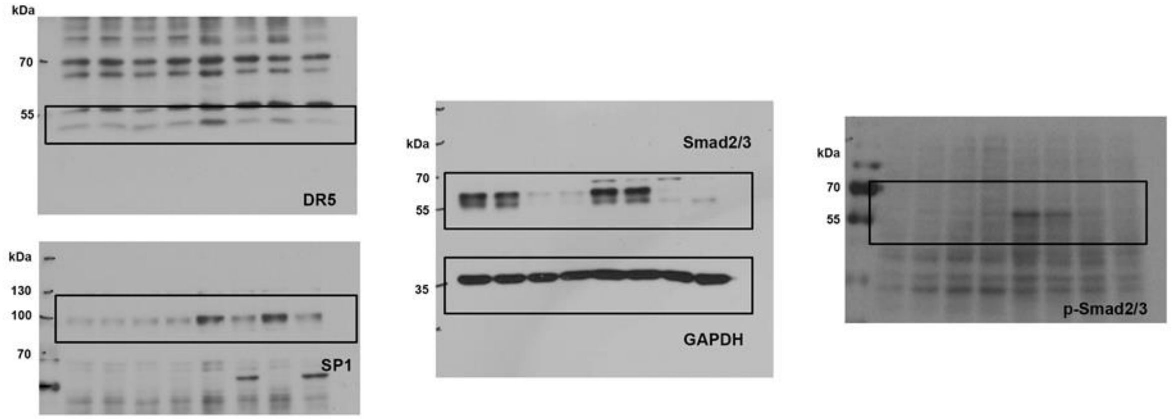

Fig. 4d

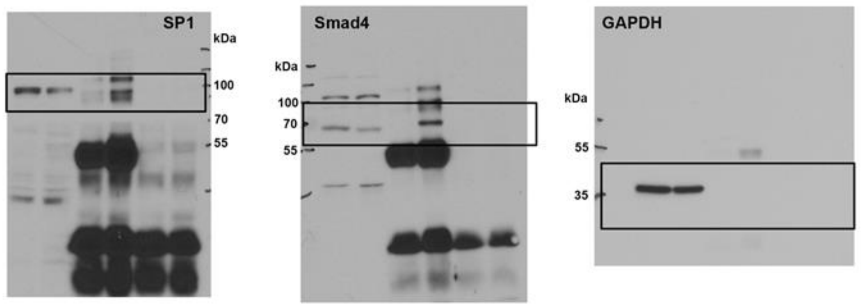

sFig.1 a

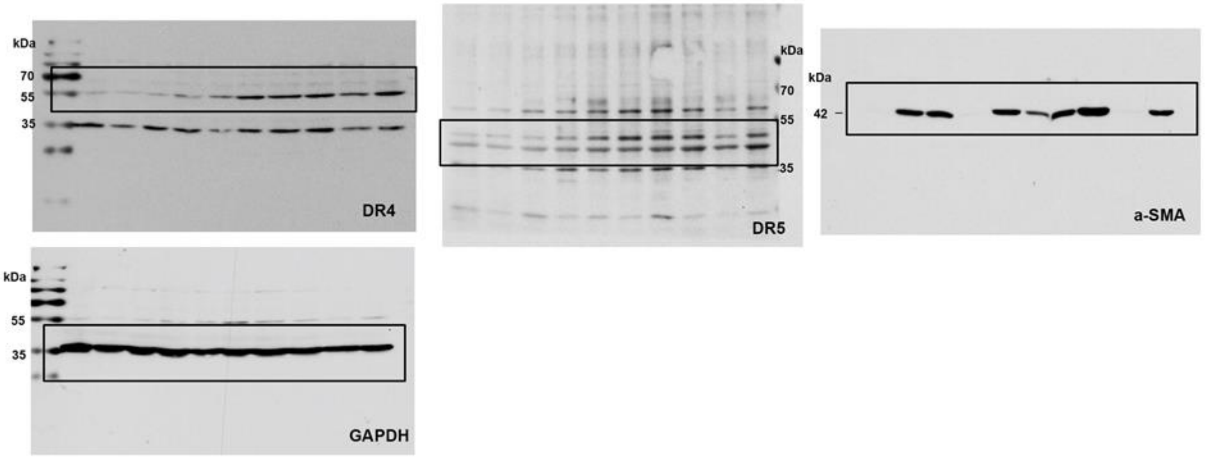

sFig.2f

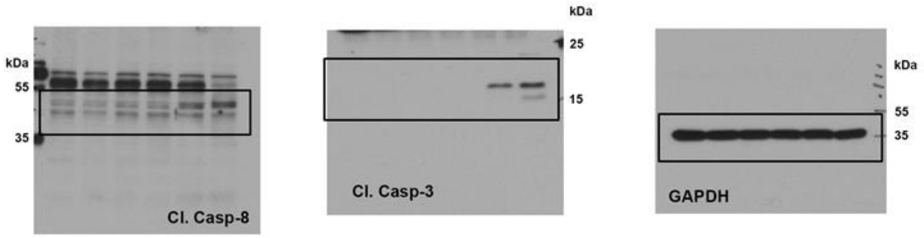

sFig.4c

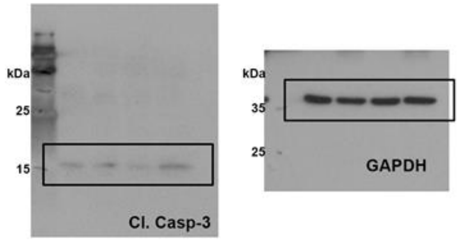

sFig.4d

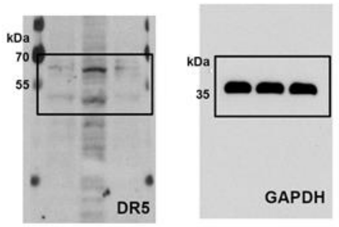

sFig. 4f

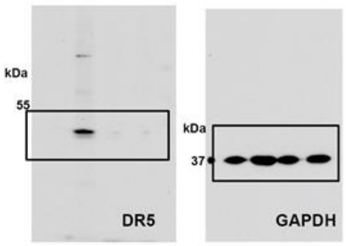

sFig. 9a

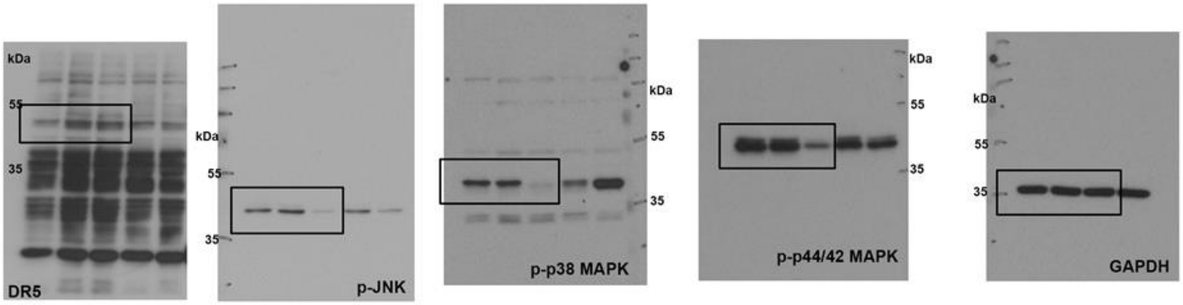

sFig. 9c

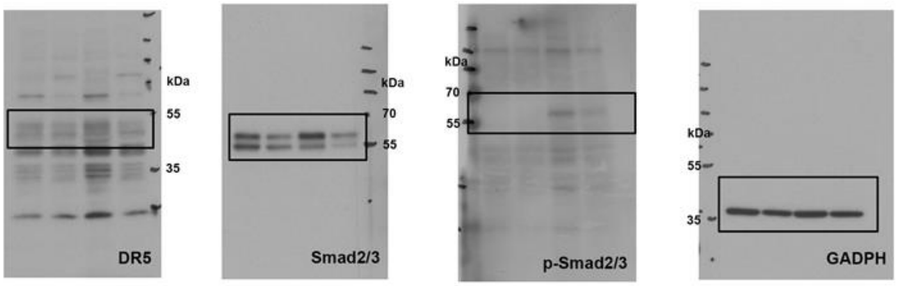

Supplement: Supplementary file 1 — Supplementary Information [file 41467_2019_9101_MOESM1_ESM.pdf]
