# Supplementary material for: Targeting of dermal myofibroblasts through death receptor 5 arrests fibrosis in mouse models of scleroderma
Source: Nat Commun. 2019 Mar 8;10:1128. doi: 10.1038/s41467-019-09101-4 (PMC6408468; doi:10.1038/s41467-019-09101-4)
Supplement: Supplementary file 2 — Description of Additional Supplementary Files [file 41467_2019_9101_MOESM2_ESM.docx]

**Description of Supplementary Files**

**File Name:** Supplementary Data 1

**Description:** Skin RNA-seq data. Data is provided in the form of raw TPM values.
