# Supplementary material for: Targeting of dermal myofibroblasts through death receptor 5 arrests fibrosis in mouse models of scleroderma
Source: Nat Commun. 2019 Mar 8;10:1128. doi: 10.1038/s41467-019-09101-4 (PMC6408468; doi:10.1038/s41467-019-09101-4)
Supplement: Supplementary file 4 — Reporting Summary [file 41467_2019_9101_MOESM4_ESM.pdf]

nature research

Corresponding author(s): Seulki Lee

Last updated by author(s): Feb 2, 2019

Reporting Summary

Nature Research wishes to improve the reproducibility of the work that we publish. This form provides structure for consistency and transparency in reporting. For further information on Nature Research policies, see Authors & Referees and the Editorial Policy Checklist.

Statistics

For all statistical analyses, confirm that the following items are present in the figure legend, table legend, main text, or Methods section.

n/a	Confirmed	
☐	☒	The exact sample size (n) for each experimental group/condition, given as a discrete number and unit of measurement
☐	☒	A statement on whether measurements were taken from distinct samples or whether the same sample was measured repeatedly
☐	☒	The statistical test(s) used AND whether they are one- or two-sided<br>*Only common tests should be described solely by name; describe more complex techniques in the Methods section.*
☐	☒	A description of all covariates tested
☐	☒	A description of any assumptions or corrections, such as tests of normality and adjustment for multiple comparisons
☐	☒	A full description of the statistical parameters including central tendency (e.g. means) or other basic estimates (e.g. regression coefficient) AND variation (e.g. standard deviation) or associated estimates of uncertainty (e.g. confidence intervals)
☐	☒	For null hypothesis testing, the test statistic (e.g. F, t, r) with confidence intervals, effect sizes, degrees of freedom and P value noted<br>*Give P values as exact values whenever suitable.*
☒	☐	For Bayesian analysis, information on the choice of priors and Markov chain Monte Carlo settings
☒	☐	For hierarchical and complex designs, identification of the appropriate level for tests and full reporting of outcomes
☒	☐	Estimates of effect sizes (e.g. Cohen's d, Pearson's r), indicating how they were calculated

Our web collection on statistics for biologists contains articles on many of the points above.

Software and code

Policy information about availability of computer code

Data collection	N/A
Data analysis	All data were analyzed using GraphPad Prism 7 software and ImageJ was used to normalize the western blotting. Data were presented as interquartile range (IQR) with at least 3 biologically independent experiments. Representative morphological images were taken out of at least 3 biologically independent experiments with similar results. An unpaired two-tailed Student's test or an two-way ANOVA test to assess the statistical significance. Assessments with p < 0.05 were considered significant.

For manuscripts utilizing custom algorithms or software that are central to the research but not yet described in published literature, software must be made available to editors/reviewers. We strongly encourage code deposition in a community repository (e.g. GitHub). See the Nature Research guidelines for submitting code & software for further information.

Data

Policy information about availability of data

All manuscripts must include a data availability statement. This statement should provide the following information, where applicable:

- Accession codes, unique identifiers, or web links for publicly available datasets
- A list of figures that have associated raw data
- A description of any restrictions on data availability

N/A

Field-specific reporting

Please select the one below that is the best fit for your research. If you are not sure, read the appropriate sections before making your selection.

☒ Life sciences ☐ Behavioural & social sciences ☐ Ecological, evolutionary & environmental sciences

For a reference copy of the document with all sections, see nature.com/documents/nr-reporting-summary-flat.pdf

Life sciences study design

All studies must disclose on these points even when the disclosure is negative.

Sample size	- Prior to starting the full-scale experiments, preliminary experiments with 5 mice per each group were performed. Based on preliminary positive experimental results, a second experiment was carried out by increasing n number.<br>- TLY012 effects in blomycin-induced skin fibrosis study: All group ≥ 7<br>- TLY012 effects in Tsk-1 transgenic mouse study: All group ≥ 8<br>- MD5-1 effects in blomycin-induced skin fibrosis study; All group ≥ 8<br>-C57BL/6 toxicity study; All group ≥ 5<br>- All experimental sample size were determined based on previously published papers (ref: 1-3)<br>- Reference:<br>1) Palumbo-Zerr et al., Nat Med. 2015;21(2):150-8<br>2) Dees et al., J Exp Med. 2011;208(5):961-72<br>3) Ponsoye et al., Ann Rheum Dis. 2016;75(12):2142-2149
Data exclusions	No data was excluded from the analyses.
Replication	All attempts at replication were successful.<br>- The results of these experiments were obtained independently by two researchers for reliability.
Randomization	- All in vitro and tissue samples were randomized.<br>- All animals were grouped randomly.
Blinding	- In all experiments, except Western blot and qPCR, the investigators were blinded to the groups and samples manner by two independent examiners.

Reporting for specific materials, systems and methods

We require information from authors about some types of materials, experimental systems and methods used in many studies. Here, indicate whether each material, system or method listed is relevant to your study. If you are not sure if a list item applies to your research, read the appropriate section before selecting a response.

Materials & experimental systems

n/a	Involved in the study	
☐	☒	Antibodies
☐	☒	Eukaryotic cell lines
☒	☐	Palaeontology
☐	☒	Animals and other organisms
☐	☒	Human research participants
☒	☐	Clinical data

Methods

n/a	Involved in the study	
☒	☐	ChIP-seq
☐	☒	Flow cytometry
☒	☐	MRI-based neuroimaging

Antibodies

Antibodies used	We provided information regarding production company, catalog number, dilution factor, etc., in the Supplementary Table 3 of the article.<br>DR4 Abcam [ab8414, 1:1000 (WB), 1:200 (IHC)], DR5 Abcam [ab8416, 1:1000 (WB), 1:200 (IHC)], α-smooth muscle actin (α-SMA) [Sigma, A2547) 1:2000 (WB), 1:400 (IHC)], Cl. Caspase-3 (Asp 175)[Cell signaling, 9664, 1:1000 (WB), 1:1000 (IHC)], Caspase-8 (1C12)[Cell signaling (9746,1:1000 (WB)], Cl. PARP-1[Cell signaling, 9541, 1:1000 (WB)], PDGFR-β1[(Santa Cruz, sc-432 1:1000 (WB)], Smad2/3 (D7G7)[Cell signaling ,8685, 1:1000 (WB), 1:100 (IP)], Smad4 (D3M6U)[Cell signaling, 38454, 1:1000 (WB)], SP1 Cell signaling [59311,1000 (WB)], p-Smad2/3[Cell signaling, 8828, 1:1000 (WB)], p-JNK(Cell signaling, 9251, 1:1000 (WB)], p-p38 MAPK[Cell signaling, 45111, 1000 (WB)], p-p44/42 MAPK[Cell signaling ,4370, 1:1000 (WB)], GAPDH[Santa Cruz ,sc-32233, 1:10000 (WB)] Secondary antibody; Rabbit IgG HRP Linked Whole Ab, [ThermoFisher (A16104), 1:5000 (WB)], Mouse IgG HRP Linked Whole Ab [ThermoFisher (31430, 1:5000 (WB)]
Validation	- The antibodies we have used were verified in the previous studies (Ref: 1-2)<br>- Reference;

1) Oh et al., Hepatology. 2016;64(1):209-23
2) Palumbo-Zerr et al., Nat Med. 2015;21(2):150-8

Eukaryotic cell lines

Policy information about cell lines

Cell line source(s)	Human primary dermal fibroblasts were purchased from the American Type Culture Collection (ATCC, PCS-201-012). Mouse dermal fibroblasts were purchased from SciencCell (#M2300-57) For human dermal fibroblasts from patient, skin biopsies from three patients were digested using dispase II (Sigma-Aldrich, St. Louis, MO, USA) with 10% heat inactivated FCS and cells were maintained in DMEM/F-12 medium.
Authentication	- We used myofibroblasts differentiated form primary human dermal fibroblasts by TGF-beta. We conducted western blot and qPCR analysis.<br>- We used mouse dermal fibroblasts for DR5 knockdown and CRISPR Knockout experiments
Mycoplasma contamination	Mycoplasma test was carried out every two weeks using the Mycoalert Mycoplasma Detection Kit (Lonza, LT07-318).
Commonly misidentified lines (See ICLAC register)	There are no commonly misidentified cell lines.

Animals and other organisms

Policy information about studies involving animals; ARRIVE guidelines recommended for reporting animal research

Laboratory animals	(1) bleomycin-induced skin fibrosis. 6-weeks-old female DBA2/J mice were obtained from the Jackson Laboratories (ME, USA). Mice received subcutaneous injections of 100ul bleomycin (0.5 mg/ml) dissolved in 0.9% NaCl in a single location of the upper back every other day for 3 or 6 weeks. TLY012 (1 or 5 mg/kg) or MD5-1 (100ug/mouse) were intraperitoneally administered after 3 weeks bleomycin injection for weeks. (2) Tight skin-1 (Tsk-1) transgenic mice. 5-weeks-old female Tsk-1 transgenic mice were obtained from the Jackson Laboratories. TLY012 was treated at the age of 5 weeks and the outcome was evaluated at age of 10 weeks.
Wild animals	N/A
Field-collected samples	N/A
Ethics oversight	All experimental procedures were followed according to the guidelines of Laboratory Animal Manual of the National Institute of Health Guide to the Care and Use of Animals, which were approved by the Johns Hopkins Medical Institute Animal Care and Use Committe

Note that full information on the approval of the study protocol must also be provided in the manuscript.

Human research participants

Policy information about studies involving human research participants

Population characteristics	Skin tissue samples were obtained form the Johns Hopkins Hospital. All the relevant information on human skin tissues was described in the Supplementary Table 2.<br>1156179613 F 64 W Control and Morphea<br>1156682343 F 61 W Control and SSc<br>1165029641 F 68 B Control and Morphea<br>1167040671 F 33 W Control and Morphea<br>1248984720 F 51 B Control and Morphea
Recruitment	All patients signed a consent form approved by the Cutaneous Translational Research Program (CTREP) of Johns Hopkins Dermatology.
Ethics oversight	Johns Hopkins Medicine

Note that full information on the approval of the study protocol must also be provided in the manuscript.

Flow Cytometry

Plots

Confirm that:

☒ The axis labels state the marker and fluorochrome used (e.g. CD4-FITC).

☒ The axis scales are clearly visible. Include numbers along axes only for bottom left plot of group (a 'group' is an analysis of identical markers).

☒ All plots are contour plots with outliers or pseudocolor plots.

☒ A numerical value for number of cells or percentage (with statistics) is provided.

Methodology

Sample preparation	Primary dermal fibroblasts were treated with or without TGF-b1 (10 ng/mL) for 54 h. Washed with Ice cold PBS 3 times and incubated with PE-conjugated DR4, DR5 or isotype IgG1 (eBioscience) for 30 min with 1 % BSA. Unlabeled cells were washed with PBS 3 times.
Instrument	BD ACCURI C6 PLUS
Software	FlowJo VX for analysis
Cell population abundance	Isotype: 10066 cells.<br>Non activated cells : 16315 cells (DR4) and 15371 cells (DR5)<br>Activated cells : 16056 cells (DR4) and 10066 cells (DR5)
Gating strategy	FSC vs. SSC, a gate was set around the cell population to exclude cell debris and cell aggregates. (16315/20000 of DR4 , 15371/20000 of DR5 for non activated vs. 16056/20000 of DR4 , 10066/20000 of DR5 for activated cells). For sample analysis, consisting of PE-DR4 and PE-DR5, the cells were replotted on histogram (Normalized to mode) v. FL2.

☐ Tick this box to confirm that a figure exemplifying the gating strategy is provided in the Supplementary Information.
